# The RpfF Acyl-ACP thioesterase of major rice pathogen *Xanthomonas oryzae* pv*. oryzae* is essential for maintenance of membrane function and virulence

**DOI:** 10.1371/journal.ppat.1014669

**Published:** 2026-10-06

**Authors:** Lizhen Luo, Zhe Hu, Mingfeng Yan, John E. Cronan, Haihong Wang

**Affiliations:** 1 Guangdong Provincial Key Laboratory for the Development Biology and Environmental Adaptation of Agricultural Organisms, College of Life Sciences, South China Agricultural University, Guangzhou, Guangdong, China; 2 Department of Microbiology, University of Illinois at Urbana-Champaign, Urbana, Illinois, United States of America; 3 Institute of Plant Protection, Jiangxi Academy of Agricultural Sciences, Nanchang, Jiangxi, China; 4 Department of Biochemistry, University of Illinois at Urbana-Champaign, Urbana, Illinois, United States of America; University of Florida Institute of Food and Agricultural Sciences, UNITED STATES OF AMERICA

## Abstract

In *Xanthomonas*, RpfF is a bifunctional enzyme having both enoyl-ACP dehydratase and acyl-ACP thioesterase activities catalyzed by a single active site. While the dehydratase activity is essential for producing quorum-sensing signals of the diffusible signal factor (DSF) family, the physiological role of the thioesterase activity remained unclear. Singh and coworkers have reported that deletion of the gene encoding RpfF (a ∆*rpfF* strain) of the rice pathogen *X*. *oryzae* pv. *oryzae* (*Xoo*) resulted in loss of membrane integrity (Singh *et al*, 2022). We report that the loss in membrane integrity is due to lack of the RpfF thioesterase activity. This was demonstrated by restoration of membrane integrity in the Δ*rpfF* strain by expression of a foreign thioesterase. Thioesterases originating in either *E. coli* or a plant enzyme fully restored virulence in rice, bacterial growth, and tolerance of membrane stress. The thioesterase overcame the defects in membrane lipid synthesis reported previously and restored the lipid components to wild-type levels. Radiolabeling revealed that fatty acid synthesis in the Δ*rpfF* strain proceeded at only one-third of the wild-type rate, which was rescued by complementation with thioesterase encoding genes. Thus, the acyl-ACP thioesterase activity of RpfF is a key regulator of *de novo* fatty acid synthesis and membrane integrity. We propose a dual-function model wherein RpfF maintains membrane lipid homeostasis via its thioesterase activity while concurrently producing DSF signals via its dehydratase activity to orchestrate quorum sensing.

## Author summary

We demonstrate that the *Xoo* RpfF enzyme serves a dual role: it produces DSF-family quorum-sensing signals and, through its acyl-ACP thioesterase activity, maintains membrane integrity. By regulating *de novo* fatty acid biosynthesis, RpfF ensures an optimal phospholipid-to-LPS ratio and a balanced fatty acid profile, both critical for membrane function. Remarkably, this membrane-protective role is itself modulated by DSF-mediated quorum sensing, enabling the pathogen to coordinate virulence factor production with rapid membrane remodeling. Such adaptive capacity enhances stress tolerance and host adaptation, ultimately maximizing infectivity. RpfF function is conserved across *Xanthomonas* species, highlighting its broad biological and agricultural significance.

## Introduction

*Xanthomonas oryzae* and *Xanthomonas campestris* are among the most economically significant phytopathogens and represent model organisms for studying plant-microbe interactions at the molecular level [1–3]. *Xanthomonas campestris* pv. *campestris* (*Xcc*) is the causative agent of black rot in cruciferous plants, whereas *Xanthomonas oryzae* pv. *oryzae* (*Xoo*) causes bacterial leaf blight, a devastating disease of rice that can decrease yields by as much as 70% [3].

Upon host infection, both *Xoo* and *Xcc* produce a range of virulence-related factors, including extracellular enzymes, exopolysaccharide (EPS), iron-chelating siderophores, plus a type III secretion system (T3SS) which collectively promote disease progression [1,4,5]. The production of these factors is primarily governed by a quorum sensing (QS) system mediated by diffusible signal factor (DSF) family signals [1,4,6]. Four DSF-family signaling molecules have been identified (Fig 1A): DSF (*cis*-11-methyl-2-dodecenoic acid), BDSF (*ci*s-2-dodecenoic acid), IDSF (*cis*-10-methyl-2-dodecenoic acid), and CDSF (*cis*, *cis*-11-methyldodeca-2,5-dienoic acid), all derived from intermediates in the canonical fatty acid synthesis (FASII) pathway [7–10]. In *Xoo* and *Xcc*, the *rpf* (regulation of pathogenicity factors) gene cluster, *rpfBFCG*, orchestrates the synthesis, perception, and turnover of DSF signals [1,4,6,11].

**Fig 1:**
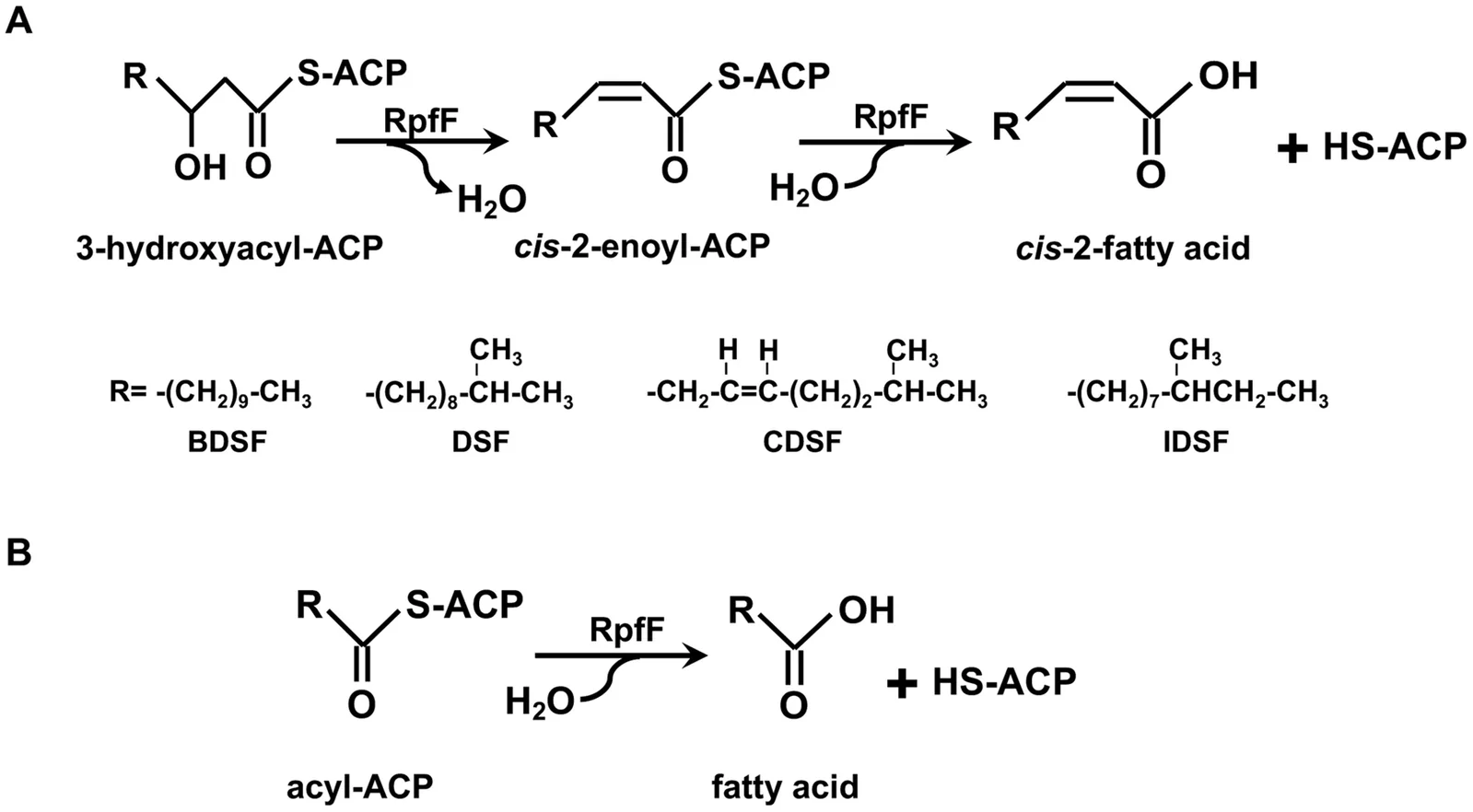
RpfF exhibits enoyl-ACP dehydratase/thioesterase activity. **(A)** Proposed pathway for DSF family signal biosynthesis. **(B)** Thioesterase activity of RpfF toward acyl-ACP substrates.

The central player in this pathway is RpfF, a bifunctional enzyme possessing both enoyl-acyl carrier protein (enoyl-ACP) dehydratase and thioesterase activities [12,13] (Fig 1). In DSF synthesis RpfF catalyzes the dehydration of 3-hydroxydodecanoyl-ACP to *cis*-2-dodecenoyl-ACP, followed by hydrolysis of the thioester bond to liberate the free fatty acid required for quorum signaling (Fig 1A) [12,13].

Intriguingly, *rpfF* mutants of *Xoo* and *Xcc* exhibit divergent phenotypes. Early studies demonstrated that DSF-deficient *Xoo rpfF* mutants retain the ability to produce extracellular enzymes and EPS but are impaired in iron uptake [14]. In contrast, *Xcc rpfF* mutants show markedly reduced production of extracellular enzymes and EPS [15,16]. Subsequent work revealed that *rpfF* in *Xoo* suppresses the production of several type II secretion system substrates, including cellulase, lipase, cellobiosidase, and xylanase [17,18], whereas in *Xcc*, RpfF promotes the production of type II effectors [19]. Furthermore, in *Xoo*, *rpfF* enhances functions related to attachment and biofilm formation while repressing motility and chemotaxis [17]; conversely, in *Xcc*, RpfF promotes biofilm dispersal [20,21]. These phenotypic differences were initially attributed to host-specific adaptations of the DSF-mediated QS circuitry in these closely related pathogens [17,21]. More recently, Singh and coworkers reported that RpfF in *Xoo* plays a major role in maintenance of membrane integrity [22]. Deletion of *rpfF* perturbs normal fatty acid biosynthesis, compromising membrane integrity and increasing permeability. This membrane damage subsequently promotes passive, non-specific leakage of extracellular enzymes (e.g., cellulase, xylanase) and heightens sensitivity to environmental stresses, collectively contributing to attenuated virulence of the Δ*rpfF* mutant. However, the precise mechanistic basis by which RpfF regulates fatty acid synthesis has remained elusive.

We report that each of the defects in membrane function reported by Singh and coworkers can be largely or totally corrected by expression of a heterologous thioesterase: the TesA thioesterase of *E. coli* [23] or the FatB1 thioesterase of the plant, *Cinnamomum camphorum* [24]. Our results establish that the acyl-ACP thioesterase activity of RpfF is indispensable for maintenance of membrane integrity and full pathogenicity of *Xoo*. Furthermore, we demonstrate that this membrane-remodeling function of RpfF is conserved across *Xanthomonas* and is dynamically regulated by the RpfC-mediated quorum-sensing circuitry, revealing a previously unrecognized integration of quorum sensing with cell membrane remodeling. These findings extend the established *Xcc* model of RpfC-mediated control of RpfF to the regulation of its thioesterase activity in *Xoo*, providing new insights into the species-specific evolution of DSF-dependent quorum sensing.

## Results

The acyl-ACP thioesterase activity of RpfF was first detected in studies of BDSF synthesis *in vitro* [25]. In attempts to demonstrate the dehydratase activity *in vitro*, the candidate acylated acyl carrier protein (ACP) substrates were hydrolyzed by thioesterase action [25] (Fig 1B). Subsequent mutagenesis studies showed that thioesterase is an intrinsic activity of both the BDSF synthetic enzyme and RpfF and that the dehydratase and thioesterase activities are catalyzed by the same active site residues [25]. Subsequent *in vitro* assays confirmed that purified RpfF hydrolyzes medium-chain acyl-ACPs (C_8_-C_14_) to release free fatty acids (FFAs) [12]. Although this activity is well-documented, its biological significance remains poorly understood. The recent report of Singh and coworkers that RpfF modulates fatty acid synthesis and maintains membrane integrity in *Xoo* [22] indicated that RpfF has physiological roles beyond DSF synthesis. We therefore hypothesized that these functions depend specifically on the RpfF acyl-ACP thioesterase activity (Fig 1B). As mentioned above the RpfF dehydratase and thioesterase activities share essential active-site residues [25], which precluded construction of separation-of-function mutants and hence we replaced the RpfF thioesterase function with heterologous enzymes. Genes encoding the acyl-CoA thioesterase TesA from *Escherichia coli* [23] and the acyl-ACP thioesterase FatB1 from the plant, *Cinnamomum camphorum* [24] were inserted into the chromosome of the *Xoo* Δ*rpfF* strain using a mini-Tn7 transposon system [26,27], generating strains Δ*rpfF*::*tesA* and Δ*rpfF*::*fatB1* (S1 Fig). This strategy allowed us to dissect the physiological contributions of the thioesterase activity independent of the dehydratase activity.

### Acyl-ACP thioesterase-encoding genes restore membrane integrity to the ∆*rpfF* strain

Singh and coworkers reported hypersensitivity of the Δ*rpfF* strain to the detergents sodium dodecyl sulfate (SDS) and Triton X-100, a characteristic of compromised membrane integrity in gram-negative bacteria [22]. To test the effects of thioesterase expression on detergent resistance we expressed *EctesA*, *CcfatB1*, or *rpfF* in the ∆*rpfF* strain. All three thioesterases restored resistance to both detergents (Fig 2A), although the CcFatB1 enzyme was somewhat more effective with Triton X-100.

**Fig 2:**
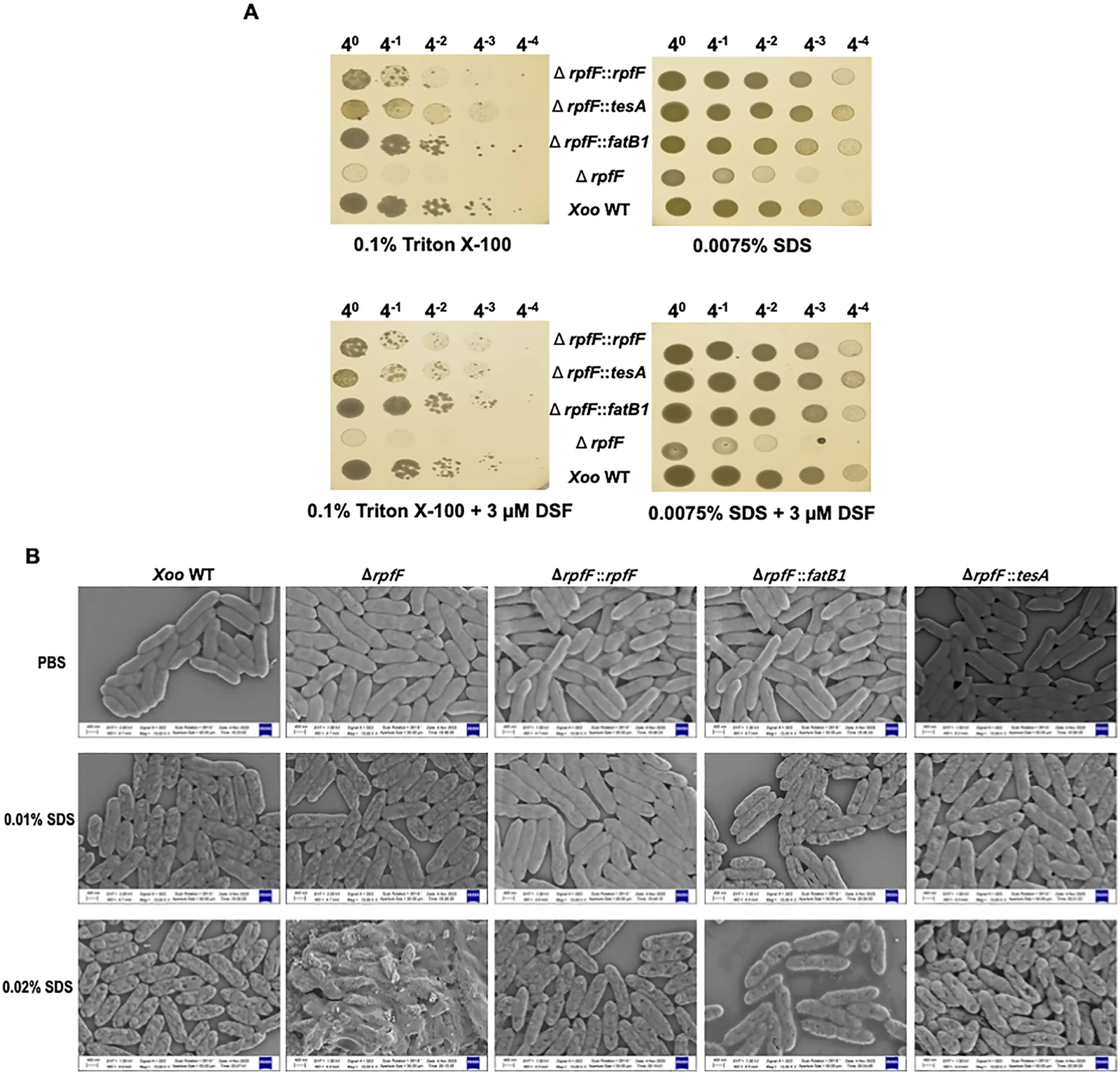
Detergent sensitivity of *Xoo rpfF* strains and its relief by thioesterase action. **(A) The Δ*rpfF* strain is sensitive to detergents**. *Xoo rpfF* strains cultures in the logarithmic phase were serially diluted and the dilution were inoculated onto NA plates containing SDS or Triton X-100 followed by incubation at 30 °C for 3 days. **(B) Scanning electron microscopy of the** ***rpfF*** **strains after SDS treatment.** The *rpfF* strains grown for 24 h were harvested, exposed to 0.01% or 0.02% SDS for 30 min, and observed by scanning electron microscopy.

Since the Δ*rpfF* strain lacks both thioesterase and DSF synthetic ability and it remained possible that DSF signals and thioesterase act synergically in ensuring *Xoo* membrane integrity. Testing of this possibility was not reported by Singh and coworkers [22], hence the effects of addition of a physiologically appropriate concentration of DSF (3 µM) on Δ*rpfF* strain detergent sensitivity were tested. Detergent sensitivity of the Δ*rpfF* strain was unaffected by DSF addition. This confirms that acyl-ACP thioesterase activity *per se* is essential for maintaining membrane integrity toward the detergents sodium dodecyl sulfate (SDS) and Triton X-100 (Fig 2A).

Scanning electron microscopy revealed that, in the absence of SDS treatment, the ∆*rpfF* strain was indistinguishable from the *Xoo* wild-type PXO99^A^ and the thioesterase expressing strains: all were rod-shaped with plump cells, uniform surfaces, and clear boundaries between cells (Fig 2B). Exposure to 0.01% SDS induced mild surface wrinkling in every strain, but the effect was most pronounced in the wild-type and the Δ*rpfF* mutant. The complemented strain Δ*rpfF*::*rpfF* retained almost native dimensions, whereas the heterologous thioesterase expressing strains Δ*rpfF*::*fatB1* and Δ*rpfF*::*tesA* showed intermediate shrinkage (Fig 2B). At 0.02% SDS, wild-type cells remained intact despite conspicuous corrugation. In stark contrast, the ∆*rpfF* strain suffered extensive cell lysis, as evidenced by the leakage of intracellular contents and the loss of distinct intercellular boundaries. Δ*rpfF*::*rpfF* and Δ*rpfF*::*tesA* resembled the wild type, whereas Δ*rpfF*::*fatB1* displayed only modest deformation (Fig 2B), indicating partial protection.

Transmission electron microscopy (TEM) was also employed to observe the morphological features of the *rpfF* strains (S2A Fig). The results indicated that upon treatment with 0.01% SDS, the cytoplasm of the ∆*rpfF* strain started to aggregate, and areas with reduced electron density emerged. In contrast, the cytoplasm of both the wild-type strain and the thioesterase-expressing strains (Δ*rpfF*::*rpfF* and Δ*rpfF*::*fatB1*) maintained a homogeneous state, while the Δ*rpfF*::*tesA* strain exhibited slight cytoplasmic aggregation. When exposed to 0.02% SDS, the cytoplasmic aggregation in the Δ*rpfF* strain became more prominent, and the areas of low electron density expanded. Although the wild-type strain and the heterologous thioesterase-expressing strains also showed responses to SDS treatment, manifested as cytoplasmic aggregation and the appearance of low-electron-density regions, the extent of these alterations was notably less significant than that observed in the Δ*rpfF* strain (S2A Fig). Among the complemented strains, Δ*rpfF*:*:fatB1* most closely resembled the wild type, while Δ*rpfF*::*rpfF* and Δ*rpfF*::*tesA* displayed intermediate phenotypes, more severe than the wild type but less severe than the Δ*rpfF* mutant (S2A Fig).

We also found that the Δ*rpfF* strain was hypersensitive to osmotic stress (addition of NaCl) and oxidative stress (H_2_O_2_ addition) (S2B Fig). These sensitivity phenotypes were suppressed by complementation with wild type *rpfF* or expression of *EctesA* whereas *CcfatB1* provided only partial rescue under these conditions (S2B Fig).

Singh and coworkers reported several other membrane integrity phenotypes in the Δ*rpfF* strain [22]. Relative to the wild type *Xoo* strain, the Δ*rpfF* strain was more permeable to two fluorescent dyes, had increased extracellular protein secretion, showed increased leakage of cytoplasmic and periplasmic proteins and induction of stress response pathways [22]. We determined the effects of thioesterase expression on each of these phenotypes. Our assays of fluorescent dye uptake in the Δ*rpfF* strain were similar to those of Singh and coworkers [22]. Thioesterase expression resulted in the decreased permeability characteristic of the wild type *Xoo* strain (S3A-S3C Fig).

The Δ*rpfF* strain had a 3- to 4-fold increase in extracellular protein secretion (Fig 3A, lane 2), consistent with its hyper-secretion of cellulase, xylanase, and lipase (Fig 3B). Complementation with *rpfF* reduced extracellular protein secretion to wild-type levels (Fig 3A, lane 5). Expression of either *EctesA* or *CcfatB1* also significantly reduced secretion, albeit less effectively than *rpfF* expression (Fig 3A, lanes 3–4). Leakage of intracellular proteins was assayed by use of enhanced green fluorescent protein (eGFP). Strains carrying an eGFP expression plasmid (pSRK-egfp) were fractionated, and eGFP was quantified by western blot. In the Δ*rpfF* mutant, the extracellular eGFP level was twice that of the wild type, while intracellular eGFP was halved, indicating severe membrane leakage (Fig 3C). Expression of *rpfF*, *EctesA*, or *CcfatB1* restored both the intracellular and extracellular eGFP fractions of the Δ*rpfF* strain to wild-type ratios (Fig 3C).

**Fig 3:**
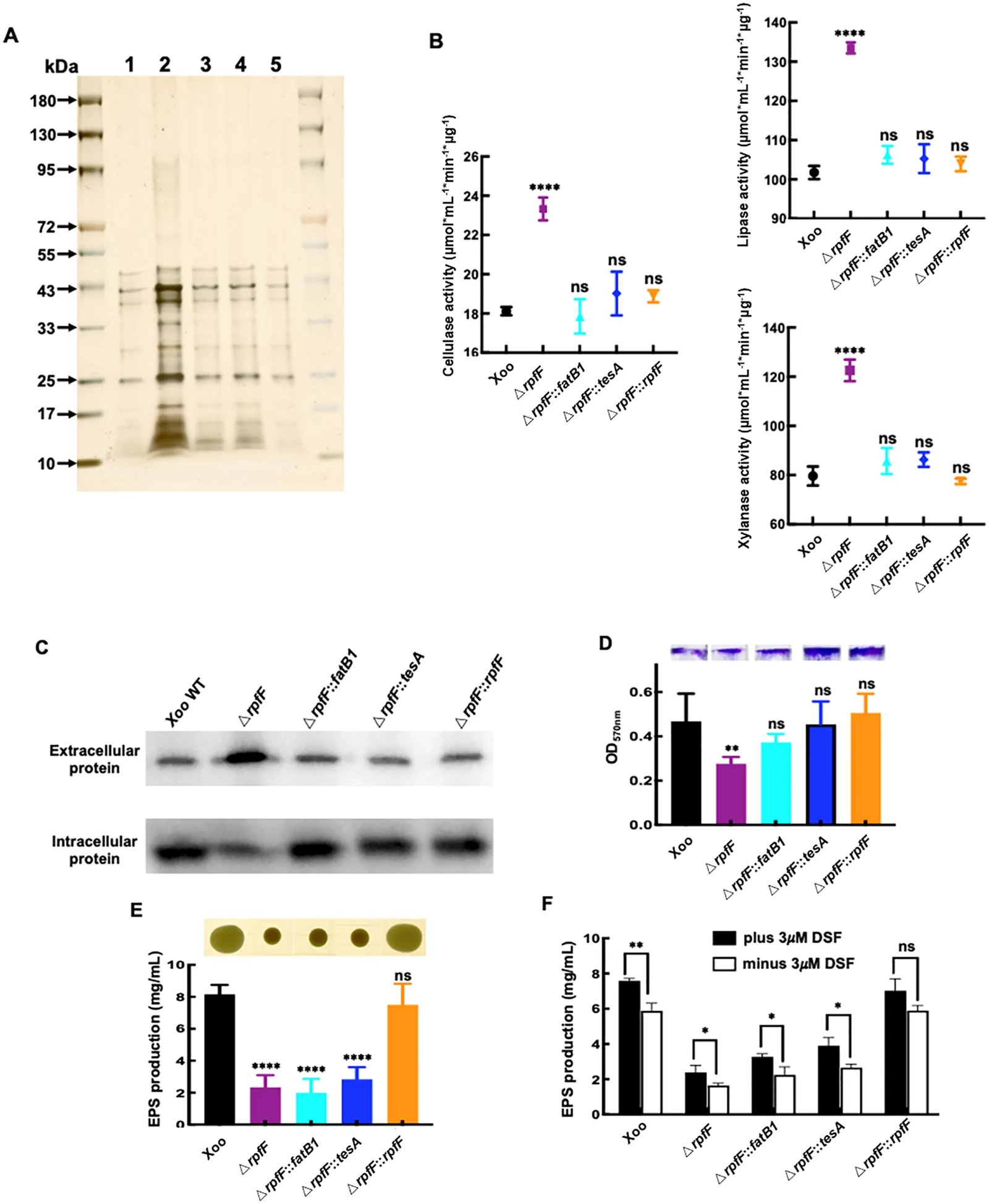
Membrane permeability profiling of the *Xoo rpfF* strains and the effects of thioesterase action. **(A) Extracellular proteins isolated from the culture supernatant of *rpfF* strains.** SDS-PAGE and silver staining were used to detect the extracellular protein of *rpfF* strains. Lane 1, *Xoo* PXO99^A^; lane 2, Δ*rpfF*; lane 3, *rpfF*::*tesA*; lane 4, *rpfF*::*fatB1*; lane 5, *rpfF*::*rpfF*. The total lane intensity (gray value) of the extracellular protein bands was measured using ImageJ software. **(B) Assessing extracellular enzyme activity in** ***Xoo rpfF*** **strains.** Cellulase, carboxymethyl cellulose (CMC) was used as substrate and activity was defined as the amount of glucose released per minute per µg of total protein per mL of reaction mixture. Xylanase, xylan was the substrate and activity was defined as the amount of xylose released per minute per µg of total protein per mL of reaction mixture. Lipases, p-nitrophenyl butyrate was the substrate and activity was defined as the amount of p-nitrophenol released per minute per µg of total protein per mL of reaction mixture. Data in B is presented as mean ± SD from n = 3 independent biological replicates. Statistical significance was determined using one-way ANOVA followed by Tukey’s multiple comparisons test. ****, *P* < 0.0001; ns, not significant. **(C) Leakage of intracellular proteins from *rpfF* strains**. The eGFP protein expression from plasmid pSRK-egfp in *rpfF* strains and the eGFP protein was detected by western blotting. **(D) Biofilm formation of** ***rpfF*** **strains**. The crystal violet method was employed to detect biofilm formation. Data in D is presented as mean ± SD from n = 6 independent biological replicates. Statistical significance was determined using one-way ANOVA followed by Tukey’s multiple comparisons test. **, *P* < 0.01; ns, not significant. **(E) EPS production of *rpfF* strains**. EPS was qualitatively detected on plates and quantitatively analyzed by the methanol precipitation method. **(F) Exopolysaccharide (EPS) production of** ***rpfF*** **strains after addition of DSF.** Strains were cultured in NA medium supplemented with 3 µM DSF; EPS was quantified from the supernatant after 48 h. Data are presented as mean ± SD of biological triplicates and analyzed by Student’s t-test. **, *P* < 0.01; *, *P* < 0.05; ns, not significant.

Consistent with defective membrane integrity, qRT-PCR analysis showed that deletion of *rpfF* up-regulated stress-response genes (*htrA*, *dsbC*, *hsp90*) and down-regulated *rpoE*. All three thioesterases restored the expression of these genes to a certain extent (S4 Fig).

We also assayed several phenotypes of the ∆*rpfF* strain that were not addressed by Singh and coworkers [22]. Biofilm formation was impaired in the ∆*rpfF* strain, showing a 39.5% reduction in biomass compared to the wild-type (Fig 3D). Complementation with *rpfF*, *EctesA* or *CcfatB1* fully restored biofilm formation (Fig 3D). Moreover, cell-aggregation assays revealed that Δ*rpfF* and Δ*rpfF*::*tesA* cells remained largely dispersed, whereas the wild-type, Δ*rpfF*::*rpfF*, and Δ*rpfF*::*fatB1* strains formed robust aggregates (S5A Fig). Deletion of *rpfF* increased swimming motility, and this hypermotile phenotype was restored to wild-type levels by genetic complementation with any of the three acyl-ACP thioesterase genes (S5B Fig). These phenotypic observations were corroborated by qRT-PCR analysis. In the Δ*rpfF* strain, transcript levels of genes encoding extracellular enzymes (*engXCA*, *xynA*) were significantly upregulated, whereas those associated with chemotaxis (*cheR*), and attachment (*yapH*, *xadA*) were markedly downregulated (S6 Fig). Complementation with *rpfF* restored the expression of these genes to near wild-type levels. Expression of *fatB1* also exhibited a substantial restorative effect, albeit to a lesser extent than *rpfF*. These findings suggest that acyl-ACP thioesterase activity suppresses extracellular enzyme secretion and bacterial motility, while promoting cell aggregation and biofilm formation.

Notably, the *rpfF* mutation led to reduced expression of *gumB*, a key gene involved in exopolysaccharide (EPS) synthesis. Complementation with *rpfF* restored *gumB* expression; however, neither *EctesA* nor *CcfatB1* was able to do so. This indicates that *gumB* expression is not regulated by the acyl-ACP thioesterase activity of RpfF and further suggests that EPS production is independent of the membrane integrity alterations resulting from the loss of this activity.

### EPS expression is unaffected by thioesterase expression and is regulated by DSF quorum sensing

The Δ*rpfF* strain exhibited an approximately fourfold reduction in EPS production compared to the wild-type strain (Fig 3E). Genetic complementation with *rpfF* fully restored EPS production in the Δ*rpfF* strain, whereas expression of *EctesA* or *CcfatB1* failed to rescue the EPS synthesis defect. This suggests that EPS synthesis is not regulated by the thioesterase activity of RpfF and is independent of the disruption in membrane integrity. We next examined the regulation of EPS synthesis by DSF signaling in *Xoo* (Fig 3F). Exogenous addition of DSF significantly increased EPS production in the wild-type strain. EPS levels were also markedly elevated in the Δ*rpfF*, Δ*rpfF*::*tesA*, and Δ*rpfF*::*fatB1* strains following DSF supplementation, although they did not reach the level observed in the wild-type strain. In the Δ*rpfF*::*rpfF* complemented strain, exogenous DSF enhanced EPS production, but the increase was not statistically significant. These findings indicate that while EPS production in *Xoo* is regulated by DSF signaling, DSF is not the only factor involved.

### Expression of heterologous thioesterases partially restores the virulence on rice of *Xoo* Δ*rpfF* strains

We assessed the effects of heterologous thioesterase expression on virulence using a leaf-clipping assay. The wild-type *Xoo* strain PXO99^A^ produced lesions averaging 17.8 ± 2.2 cm in length at 14 days post-inoculation (dpi) (Fig 4A). In contrast, the Δ*rpfF* strain produced lesions of only 2.1 ± 0.6 cm, representing an ~ 88% reduction. Introduction of the native *rpfF* gene into the Δ*rpfF* strain (Δ*rpfF*::*rpfF*) restored lesion length to 13.3 ± 2.7 cm, somewhat less than wild-type levels. Remarkably, expression of either heterologous thioesterase, EcTesA or CcFatB1, substantially restored virulence, yielding lesions of 12.5 ± 2.5 cm and 10.1 ± 2.5 cm, respectively (Fig 4A). Time-course analyses revealed congruent kinetics of lesion development: lesions expanded rapidly in the wild-type and complemented strains but progressed only slowly in the Δ*rpfF* strain (Fig 4B). Bacterial population counts per unit lesion length were only marginally lower in the mutant strain (Fig 4C), indicating that the virulence defect is impaired tissue colonization rather than *Xoo* growth deficiency in planta. Hence, acyl-ACP thioesterase activity, irrespective of its source, is sufficient to largely restore *Xoo* pathogenicity.

**Fig 4:**
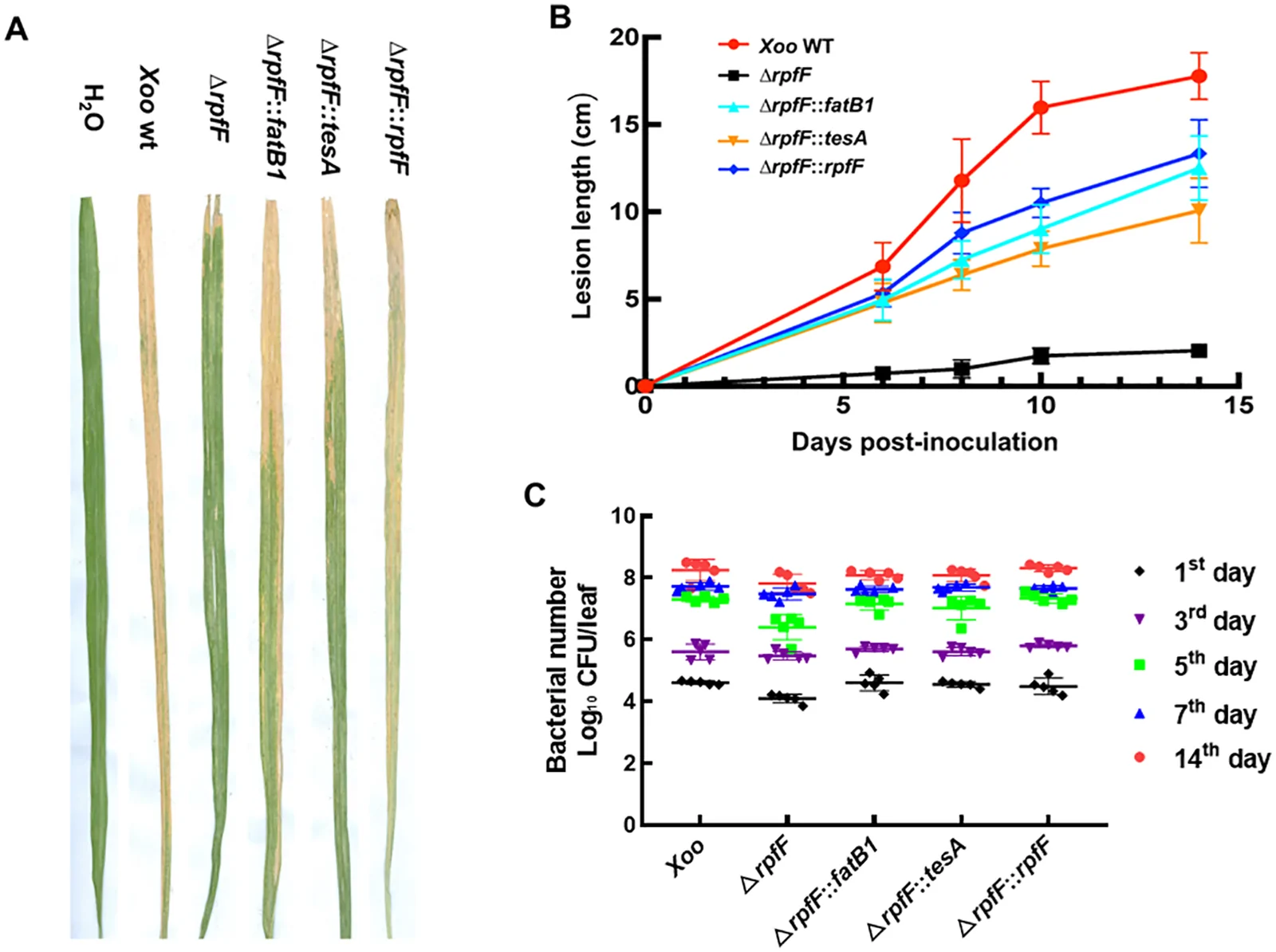
Virulence of *Xoo rpfF* mutants in rice and effects of thioesterase action. **(A) Representative leaves after clip-inoculation with the indicated *rpfF* strains. (B) Time-course of lesion expansion. (C) Bacterial population in planta.** Approximately 10^9^ cells were used to clip inoculate 40- to 45-day-old susceptible rice plants. The lesion length was then measured. The cfu values were determined from approximately 1 cm^2^ of the leaf by the serial dilution.

### RpfF acyl-ACP thioesterase modulates fatty acid synthesis to maintain *Xoo* membrane homeostasis

Singh and coworkers suggested that deletion of *rpfF* altered lipid composition of the *Xoo* cell envelope [22]. However, the data reported where only qualitative in nature and suffered from a lack of resolution of the membrane phospholipid species and no analysis of the phospholipid fatty acyl chains. We have analyzed the lipopolysaccharide (LPS), phospholipid, and phospholipid acyl chains in the ∆*rpfF* strain and the ∆*rpfF* strains expressing a thioesterase.

To profile the phospholipid composition, cultures were pulse-labeled with sodium [^14^C]acetate. Phospholipids were separated by thin-layer chromatography (TLC) and detected by autoradiography. *Xoo* membranes contain phosphatidylethanolamine (PE), phosphatidylglycerol (PG), and cardiolipin (CL), with PE predominating (Fig 5A). The Δ*rpfF* strain accumulated excess phospholipid, particularly PE and CL (Fig 5A, lane 2). The Δ*rpfF* phospholipid phenotype, was reversed by complementation with *rpfF* and by expression of *EctesA* or *CcfatB1* (Fig 5A, lanes 3–5), underscoring the role of RpfF acyl-ACP thioesterase in maintaining envelope homeostasis.

**Fig 5:**
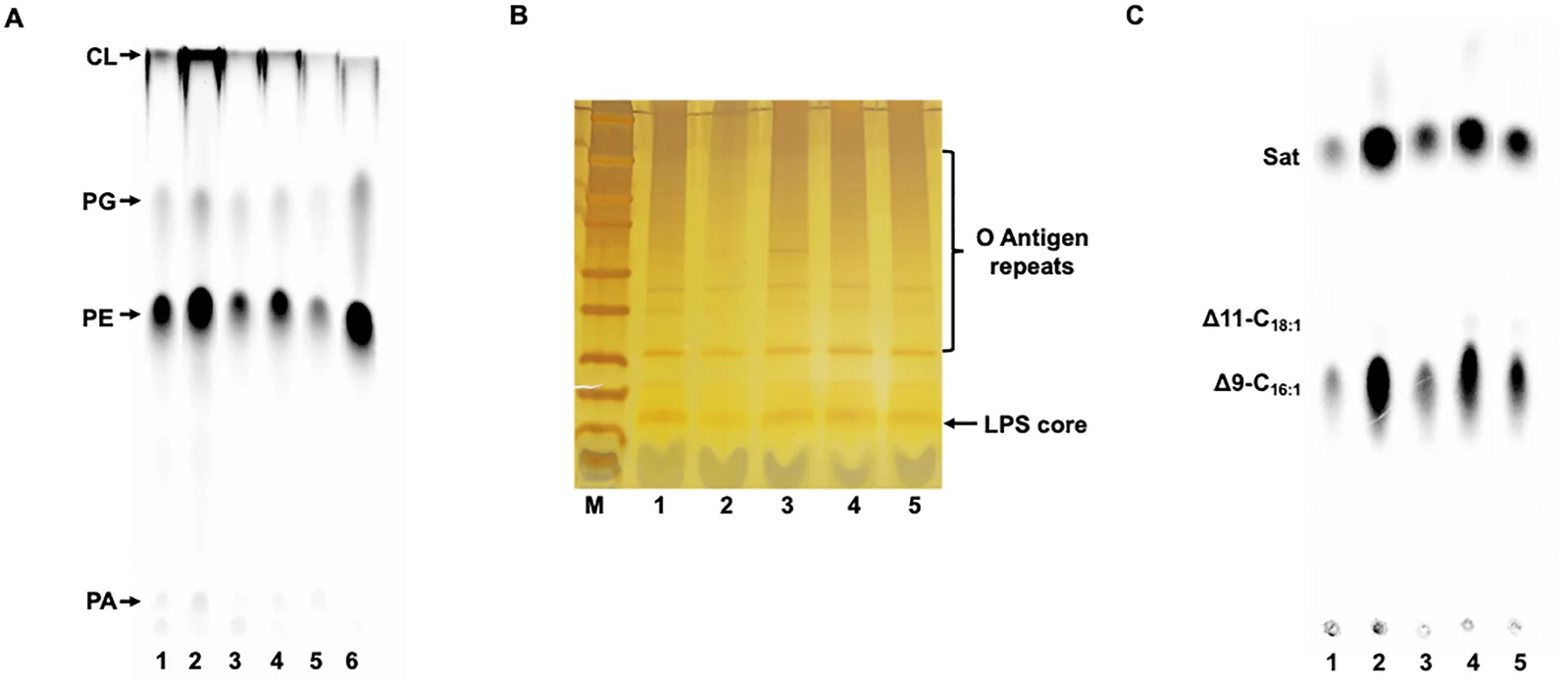
Phospholipid and lipopolysaccharide (LPS) profiles, and fatty acid synthesis efficiency, of *rpfF* strains. **(A) Phospholipid composition**. The *rpfF* strains were labeled with sodium [^14^C]sodium acetate and the phospholipids of cells was separated using thin-layer chromatography (TLC). Lane 1, strain *Xoo* PXO99^A^; lane 2, ∆*rpfF* mutant; lane 3, *rpfF*::*tesA* strain; lane 4, *rpfF*::*fatB1* srain; lane 5, *rpfF*::*rpfF* strain; lane 6, *E.coli* MG1655. **(B) Lipopolysaccharide profiles.** Lipopolysaccharide was extracted with the hot aqueous phenol, separated by SDS-PAGE and silver-stained. Lane 1, strain *Xoo* PXO99^A^; lane 2, ∆*rpfF* mutant; lane 3, *rpfF*::*tesA* strain; lane 4, *rpfF*::*fatB1* srain; lane 5, *rpfF*::*rpfF* strain*.* **(C) Fatty acid synthesis efficiency.** Cultures were grown to the logarithmic phase, normalized to the same optical density (OD_600_), and labeled with sodium [^14^C]acetate. Fatty acids were then extracted, converted to methyl esters, and separated by argentation TLC for autoradiography. Lane 1, ∆*rpfF* mutant; lane 2, strain *Xoo* PXO99^A^; lane 3, *rpfF*::*tesA* strain; lane 4, *rpfF*::*fatB1* srain; lane 5, *rpfF*::*rpfF* strain. Sat, saturated fatty acids; ∆11C_18:1_, cis-vaccenic acid; ∆9C_16:1_, palmitoleic acid.

Gas Chromatography-Mass Spectrometry (GC-MS) analysis of *Xoo* membrane phospholipids revealed that the acyl chains comprised saturated, unsaturated, and branched-chain species, consistent with profiles observed in other *Xanthomonas* species [7,9] (Table 1). The ∆*rpfF* strain exhibited altered levels of these fatty acids, with significant changes specifically in C_16:0_, C_16:1_, and *iso*-C_17:0_. Specifically, the C_16:1_ content increased from 29.66% ± 0.58% in the wild-type strain to 33.69% ± 1.2% in the ∆*rpfF*, whereas the levels of C_16:0_ and *iso*-C_17:0_ decreased from 34.99% ± 0.52% and 20.69% ± 0.85% to 30.96% ± 2.27% and 16.91% ± 1.53%, respectively (Table 1). Expression of either *EctesA* or *CcfatB1* partially restored the fatty acid levels to those of the wild-type strain. Although complementation with *rpfF* alone did not restore the individual levels of C_16:0_ and *iso*-C_17:0_ in the the ∆*rpfF*, the combined levels of these two fatty acids were essentially equivalent to those of the wild-type strain. Collectively, these findings indicate that the *rpfF* mutation alters the ratio of unsaturated (C_16:1_) to saturated (C_16:0_ and iso-C_17:0_) fatty acids in membrane phospholipids, and this imbalance is corrected by genetic complementation with *rpfF* or by expression of either *EctesA* or *CcfatB1*. These results demonstrate that the altered membrane composition is a direct consequence of the loss of RpfF acyl-ACP thioesterase activity.

**Table 1:** Fatty acid compositions of total lipid extracts from *Xoo* mutant strains^a^.

Fatty acid^b^		% Of the total fatty acids			
	*Xoo* WT	ΔrpfF**	Δ*rpfF*::*fatB1*	Δ*rpfF*::*tesA*	Δ*rpfF*::*rpfF*
n-C_14:0_	0.43 ± 0.09	0.8 ± 0.08	0.37 ± 0.06	0.47 ± 0.05	0.46 ± 0.09
*iso*-C_15:0_	3.69 ± 0.13	5.68 ± 0.49	3.86 ± 0.32	4.29 ± 0.06	5.31 ± 0.57
n-C_15:0_	0.42 ± 0.03	0.53 ± 0.18	0.32 ± 0.04	0.56 ± 0.03	0.57 ± 0.04
n-C_16:1_	29.66 ± 0.58	33.69 ± 1.2	29.87 ± 1.01	29.74 ± 0.9	27.58 ± 0.74
n-C_16:0_	34.99 ± 0.52	30.96 ± 2.27	36.71 ± 1.34	34.28 ± 0.22	30.85 ± 0.58
*iso*-C_17:0_	20.69 ± 0.85	16.91 ± 1.53	19.66 ± 0.5	21.57 ± 1.01	24.65 ± 1.07
n-C_17:1_	2.09 ± 0.02	4.11 ± 3.49	1.79 ± 0.1	2.18 ± 0.03	2.58 ± 0.11
n-C_18:1_	5.91 ± 0.2	4.72 ± 1.05	5.68 ± 0.21	4.81 ± 0.24	5.51 ± 0.12
n-C_18:0_	1.25 ± 0.44	1.89 ± 0.35	0.96 ± 0.12	1.18 ± 0.11	1.15 ± 0.12

^a^Cells were grown in NA medium at 30 °C for 36 h. The total lipids were extracted and trans-esterified to obtain fatty acid methyl esters, and the products were identified using GC-MS. The values are percentages of total fatty acids and are presented as the mean ± standard deviation of three independent experiments. All statistical comparisons were performed between each mutant or complemented strain and the wild-type strain *Xoo* PXO99^A^ using Student’s t-test. Asterisks indicate significant differences compared with *Xoo* WT: **, *P* < 0.01. Unmarked strains are not significantly different from *Xoo* WT (*P* > 0.05). ^b^n-C_14:0_, myristic acid; 3-OH-C_14:0_, 3-hydroxytetradecanoic acid; *iso*-C_15:0_, 13-methyl-tetradecanoic acid; n-C_16:0_, palmitic acid; n-C_16:1_, palmitoleic acid; *iso*-C_17:0_, 15-methyl-palmitic acid; n-C_18:0_, octadecenoic acid; n-C_18:1_, *cis*-11-octadecenoic acid.

LPS was extracted with hot aqueous phenol, resolved by SDS-PAGE, and visualized by silver staining. The LPS profile of the *rpfF* deletion mutant resembled that of the wild-type strain but showed markedly lower intensity for both the O-antigen repeats and the core oligosaccharide (Fig 5B, lanes 1–2). The expression of *rpfF*, *EctesA*, or *CcfatB1* in ∆*rpfF* strain restored LPS abundance to wild-type levels (Fig 5B, lanes 3–5). Lipid A-to-phospholipid ratios of *rpfF* strains were also quantified by scintillation counting (Table C in S1 Appendix). The wild-type ratio was 0.117, whereas the ∆*rpfF* strain dropped to 0.037. Re-introducing *rpfF* restored the ratio to 0.133 whereas expression of *EctesA* elevated it further to 0.175, while *CcfatB1* yielded only 0.060, markedly less effective than either *rpfF* or *EctesA*. These data indicate that loss of *rpfF* skews the lipid A/phospholipid balance in *Xoo* and that thioesterase activity can compensate for this defect.

Since RpfF hydrolyzes acyl-ACPs to release FFAs [12,13], we investigated whether it regulates *de novo* fatty acid synthesis by altering the cellular pool of acyl-ACP intermediates. Cells were labeled with [^14^C]acetate, and the resulting fatty acid methyl esters were resolved by argentation-TLC and quantified. Radiolabel incorporation into fatty acids in the Δ*rpfF* mutant strain was only 12% of the wild-type level (Fig 5C, lanes 1–2) whereas expression of *rpfF*, *EctesA*, or *CcfatB1* increased incorporation to 33%, 27% and 56% of wild-type levels, respectively (Fig 5C, lanes 3–5). Scintillation counting yielded specific synthesis rates (cpm/min) of 414.87 ± 0.50 (wild type), 145.26 ± 0.56 (Δ*rpfF*), 355.44 ± 1.38 (Δ*rpfF*::*tesA*), 318.69 ± 0.69 (Δ*rpfF*::*fatB1*), and 363.52 ± 1.05 (Δ*rpfF*::*rpfF*). These data firmly establish the acyl-ACP thioesterase activity of RpfF is a pivotal regulator of fatty acid biosynthetic flux in *Xoo*.

### *In vitro* and *in vivo* characterization of RpfF thioesterase activity

In the above experiments we observed some differences in phenotype restoration by the RpfF, EcTesA and CcFatB1 thioesterases that could be explained by different acyl chain specificities. To assess the specificities acyl-ACPs with C_4_-C_14_ acyl chains were first generated with *Vibrio harveyi* acyl-ACP synthetase (AasS) and purified to homogeneity. Acyl-ACP thioesterase activities were then monitored by conformationally sensitive gel electrophoresis, which resolves the *holo*-ACP product from its acylated precursor. When incubated with butyryl-ACP (C_4_) (Fig 6A, lanes 1–4) or hexanoyl-ACP (C_6_) (lanes 6–9), RpfF, EcTesA, and CcFatB1 generated only trace amounts of *holo*-ACP, indicating weak activity against short-chain substrates. Activity increased substantially with octanoyl-ACP (C_8_) (Fig 6B, lanes 1–4) and decanoyl-ACP (C_10_) (lanes 6–9). All three enzymes completely hydrolyzed dodecanoyl-ACP (C_12_) (Fig 6C, lanes 1–4) and tetradecanoyl-ACP (C_14_) (Fig 6C, lanes 6–9) to *holo*-ACP. Thus, RpfF, EcTesA and CcFatB1, have similar chain-length-dependent acyl-ACP thioesterase activities. We next quantified substrate activity using a *holo*-ACP thiol release assay. RpfF hydrolyzed acyl-ACPs across the entire C_4_-C_14_ range, but its chain-length preference and catalytic efficiency differed from those of EcTesA and CcFatB1 (S7A Fig).

**Fig 6:**
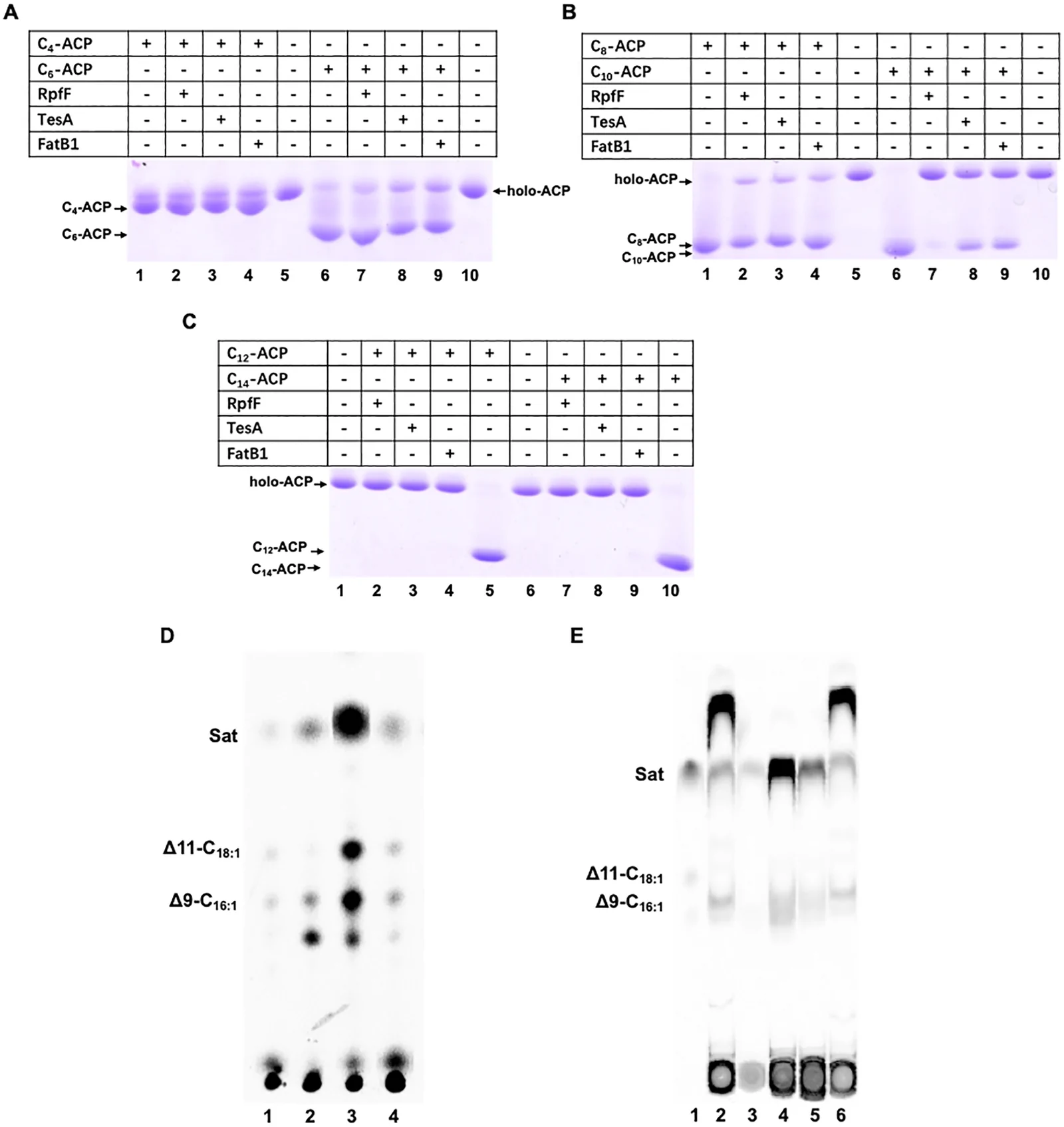
Characterization of RpfF acyl-ACP thioesterase activity. **(A-C) RpfF acyl-ACP thioesterase activity** ***in vitro*.** Reactions (30 min at 37 °C) contained 20 µM acyl-ACP, and 0.2 µg purified enzyme (RpfF, TesA, or FatB1), products were resolved on urea-PAGE gels and stained with Coomassie Brilliant Blue R-250. **(A) Short-chain substrates.** Lane 1-4, butyryl-ACP(C_4_-ACP) used as substrate; lane 2-4, RpfF, TesA or FatB1 used as acyl-ACP thioesterase respectively. Lane 6-9, hexanoyl-ACP (C_6_-ACP) used as substrate; lane 7-9, RpfF, TesA or FatB1 used as acyl-ACP thioesterase respectively. Lane 5 and 10, *holo*-ACP as control. **(B) Medium-chain substrates.** Lane 1-4, octanoyl-ACP (C_8_-ACP) used as substrate; lane 2-4, RpfF, TesA or FatB1 used as acyl-ACP thioesterase respectively. Lane 6-9, decanoyl-ACP (C_10_-ACP) used as substrate; lane 7-9, RpfF, TesA or FatB1 used as acyl-ACP thioesterase respectively. Lane 5 and 10, *holo*-ACP as control. **(C) Long-chain substrates.** Lane 1-4, dodecanoyl-ACP (C_12_-ACP) used as substrate; lane 2-4, RpfF, TesA or FatB1 used as acyl-ACP thioesterase respectively. Lane 6-9, tetradecanoyl-ACP (C_14_-ACP) used as substrate; lane 7-9, RpfF, TesA or FatB1 used as acyl-ACP thioesterase respectively. Lane 5 and 10, *holo*-ACP as control. **(D-E) RpfF acyl-ACP thioesterase activity *in vivo*.** The strains were then labeled with [^14^C]acetate, and the free fatty acid contents of culture supernatants was analyzed using argentation thin-layer chromatography. **(D) Free fatty acids production in *E. coli fadD* background.** Lane 1, free fatty acids from *E. coli fadD* mutant carrying empty vector; lane 2, free fatty acids from *E. coli fadD* mutant carrying pSRK-tesA; lane 3, free fatty acids from *E. coli fadD* mutant carrying pSRK-fatB1; lane 4, free fatty acids from *E. coli fadD* mutant carrying pSRK-rpfF. **(E) Free fatty acids production in** ***Xoo rpfF*** **strains.** Lane 1, fatty acids from *E. coli* strain MG1655 used as control; lane 2, free fatty acids from *Xoo ∆rpfB ∆rpfC* double mutant; lane 3, free fatty acids from *Xoo ∆rpfB ∆rpfC ∆rpfF* triple mutant; lane 4, free fatty acids from *Xoo ∆rpfB ∆rpfC ∆rpfF*::*tesA* strain; lane 5, free fatty acids from *Xoo ∆rpfB ∆rpfC ∆rpfF*::*fatB1* strain; lane 6, free fatty acids from *Xoo ∆rpfB ∆rpfC ∆rpfF*::*rpfF* strain.

To provide an assay for *in vivo* acyl-ACP thioesterase activity of the thioesterases, we expressed *rpfF*, *EctesA*, or *CcfatB1* from the pSRK-Km plasmid in the *E. coli fadD* strain JW1794 [25]. The *fadD* mutation blocks the fatty acid degradation pathway allowing free fatty acids generated by acyl-ACP thioesterase action to accumulate extracellularly [24,25,28]. After labeling cells with [^14^C]acetate, we analyzed culture supernatants by argentation-TLC. The *rpfF*-bearing strain released free fatty acids (Fig 6D, lane 4), but at levels substantially lower than strains expressing *EctesA* or *CcfatB1* (Fig 6D, lanes 2–3). Moreover, the free fatty acids (FFAs) profile of the *rpfF*-bearing strain was distinct, underscoring unique substrate specificity.

In *Xcc*, deletion of *rpfF* abolishes both FFA secretion and DSF family signals synthesis [12,29]. To determine whether *Xoo* RpfF has a similar role, we compared the fatty acid secretion of the *Xoo* ∆*rpfB* ∆*rpfC* double mutant strain with that ∆*rpfB* ∆*rpfC* ∆*rpfF* triple mutant strain, each expression of *rpfF*, *EctesA*, or *CcfatB1* (Fig 6E). The double mutant secreted abundant saturated and unsaturated FFAs (Fig 6E, lane 2), whereas the triple mutant produced only traces of saturated species (Fig 6E, lane 3). Complementation with *rpfF* restored secretion to levels comparable to the double mutant (Fig 6E, lane 6). In contrast, expression of *EctesA* or *CcfatB1* yielded even higher saturated fatty acid levels (Fig 6E, lanes 4–5), again highlighting the distinct chain-length preferences of RpfF.

We also quantified DSF-family signals in a ∆*rpfB* ∆*rpfC* strain, this background, constitutively overproduces these DSF compounds due to disrupted signal turnover and regulation [4]. The triple mutant ∆*rpfB* ∆*rpfC* ∆*rpfF* produced no detectable CDSF, BDSF, IDSF, or DSF (S7B Fig). In contrast although ectopic expression of *rpfF* fully restored DSF production to Δ*rpfB* Δ*rpfC* levels, neither *EctesA* nor *CcfatB1* enabled production of any DSF-family signals (S7B Fig). This finding unequivocally demonstrates that the virulence defect of the Δ*rpfF* mutant is not solely due to the loss of QS signaling but is primarily a consequence of the absence of acyl-ACP thioesterase activity.

## Discussion

Although RpfF has been known to possess acyl-ACP thioesterase activity for over a decade [12,13], its physiological role beyond DSF signals biosynthesis has remained largely enigmatic. This study provides compelling genetic and biochemical evidence that the acyl-ACP thioesterase activity of RpfF is indispensable for maintaining *Xoo* membrane homeostasis and is a critical determinant of full virulence of this bacterium.

Replacement of the RpfF thioesterase in the Δ*rpfF* strain with two phylogenetically distinct acyl-ACP thioesterase encoding genes (*EctesA* and *CcfatB1*) restored wild-type growth, stress tolerance, and pathogenicity on rice. Remarkably, the complemented strains regained the ability to produce major virulence factors except exopolysaccharide (EPS). This selective restoration indicates that EPS production is exclusively controlled by the DSF-dependent QS circuit, whereas the other phenotypes are modulated by the acyl-ACP thioesterase activity of RpfF.

For Gram-negative bacterial pathogens, the ability to adapt to environmental stress, particularly during host infection, is critically dependent on extensive membrane remodeling [30,31]. This process involves coordinated changes in phospholipids, LPS, and membrane-associated proteins [32–35]. In Gram-negative bacteria, membrane integrity is governed by the stoichiometric balance between phospholipids and LPS, as well as by the precise acyl-chain composition of the lipids [36–39]. Deletion of *rpfF* disrupted this delicate balance: phospholipid content increased while LPS levels declined, and unsaturated fatty acids accumulated at the expense of long-chain saturated and branched-chain species. Expression of acyl-ACP thioesterase genes re-established both the phospholipid-to-LPS ratio and the wild-type fatty acid profile.

The mechanistic basis for these compositional shifts is a severe impairment in *de novo* fatty acid synthesis in the *rpfF* deletion mutant. [^14^C]acetate labeling revealed that the *rpfF* deletion mutant synthesizes fatty acids at only one-third the wild-type rate, whereas expression of acyl-ACP thioesterase genes restored synthetic flux to near-wild-type levels. We therefore propose that the acyl-ACP thioesterase activity of RpfF relieves acyl-ACP-mediated feedback inhibition of the FASII pathway, thereby sustaining the supply of fatty acid acyl groups required for membrane biogenesis and homeostasis.

The membrane-remodeling function of the RpfF acyl-ACP thioesterase is not unique to *Xoo* but exhibits conservation across *Xanthomonas*. Deletion of *rpfF* in *Xcc* likewise conferred hypersensitivity to detergent and induced a pronounced shift in the phospholipid-to-LPS ratio (S8 Fig), and genetic re-introduction of an acyl-ACP thioesterase-encoding allele fully restored membrane integrity in the *Xcc* Δ*rpfF* mutant.

The binding of RpfC to RpfF and the consequent inhibition of its activity were first discovered and mechanistically characterized in *Xcc*. Previous genetic and biochemical studies have established that the REC domain of RpfC physically interacts with RpfF, thereby negatively regulating DSF-family signal biosynthesis [40,41]. Structural analyses further revealed that the REC domain of RpfC locks RpfF in an inactive conformation by physically blocking the entrance to its substrate-binding pocket, providing a direct mechanistic link between the quorum-sensing apparatus and RpfF enzymatic activity [41]. Current models propose that the sensor kinase RpfC exerts dual control over DSF signaling [1,4,40,41]. Upon perceiving DSF family signals, RpfC phosphorylates the response regulator RpfG to activate virulence gene expression [1,4]. Concurrently, RpfC physically interacts with RpfF via its REC domain, thereby modulating RpfF enzymatic activity [40,41]. This regulatory interaction is recognized as a core component of DSF-family signal-mediated quorum sensing in *Xcc*.

Our *in* *vivo* and *in* *vitro* data demonstrate that this regulatory mechanism is conserved in *Xoo*. Overexpression of RpfC in wild-type *Xoo* phenocopied the detergent hypersensitivity of the Δ*rpfF* mutant (S9A Fig), suggesting that excess RpfC sequesters and inhibits endogenous RpfF activity. Consistent with this, the isolated REC domain of RpfC suppressed the acyl-ACP thioesterase activity of purified RpfF *in* *vitro* (S9B Fig). These findings extend the established *Xcc* model, in which RpfC negatively regulates RpfF to control DSF production, to the regulation of RpfF’s thioesterase activity in *Xoo*. Collectively, these observations indicate that the DSF quorum-sensing circuit dynamically modulates fatty acid biosynthesis and, consequently, membrane architecture, and that this regulatory paradigm extends from the established *Xcc* model to *Xoo*.

The divergent *rpfF* mutant phenotypes between *Xoo* and *Xcc*, as described in the Introduction, were initially attributed to host-specific adaptations of the DSF-mediated QS circuitry [14–21]. Using an osmoprotectant (5% sucrose) rescue experiment, Singh *et al*. demonstrated that the increased extracellular enzyme activity in the *Xoo rpfF* mutant is largely due to passive, non-specific leakage through a compromised membrane, rather than active T2SS-mediated secretion or transcriptional activation via the DSF/c-di-GMP/Clp pathway [22]. Our heterologous thioesterase complementation results directly support and extend this conclusion: restoring membrane integrity (via thioesterase activity) reduces extracellular enzyme leakage to wild-type levels, even though DSF signaling is not restored. Thus, the species-specific outcomes of *rpfF* deletion on extracellular enzyme production arise not from a difference in the core RpfF thioesterase function, which is conserved, but from the interplay between the conserved membrane-maintenance function of RpfF, which, when disrupted, causes passive leakage of enzymes in *Xoo*, and the divergent DSF-dependent QS circuitry that, in *Xcc*, transcriptionally regulates T2SS effector production.

Our data lead to the proposal that *Xanthomonas* integrates QS with cell membrane remodeling. As cell density increases, the accumulation of DSF family species relieves RpfC-mediated inhibition of RpfF, thereby accelerating fatty acid production and reconfiguring the membrane to withstand host-imposed stresses. This regulatory network allows the pathogen to synchronize virulence factor production with adaptive membrane restructuring, optimizing survival and proliferation within the host environment.

In integrating these findings, we present a dual-function model for RpfF (Fig 7). The enzyme embodies two catalytic activities within a single polypeptide: (i) an enoyl-ACP dehydratase activity that generates the DSF family quorum-sensing signals, and (ii) an acyl-ACP thioesterase activity that regulates the intracellular acyl-ACP pool and, consequently, the rate of fatty acid synthesis. Through its thioesterase activity, RpfF maintains the stoichiometry of phospholipids and LPS and fine-tunes the acyl-chain composition of the membrane, thereby preserving membrane integrity. Loss of *rpfF* simultaneously abolishes quorum-sensing and disrupts membrane homeostasis, leading to pleiotropic defects in growth, stress tolerance, and virulence. While we acknowledge that the integration of quorum sensing with cell membrane remodeling is supported by convergent lines of evidence from both *Xcc* and *Xoo*, we also recognize that further mechanistic details remain to be elucidated.

**Fig 7:**
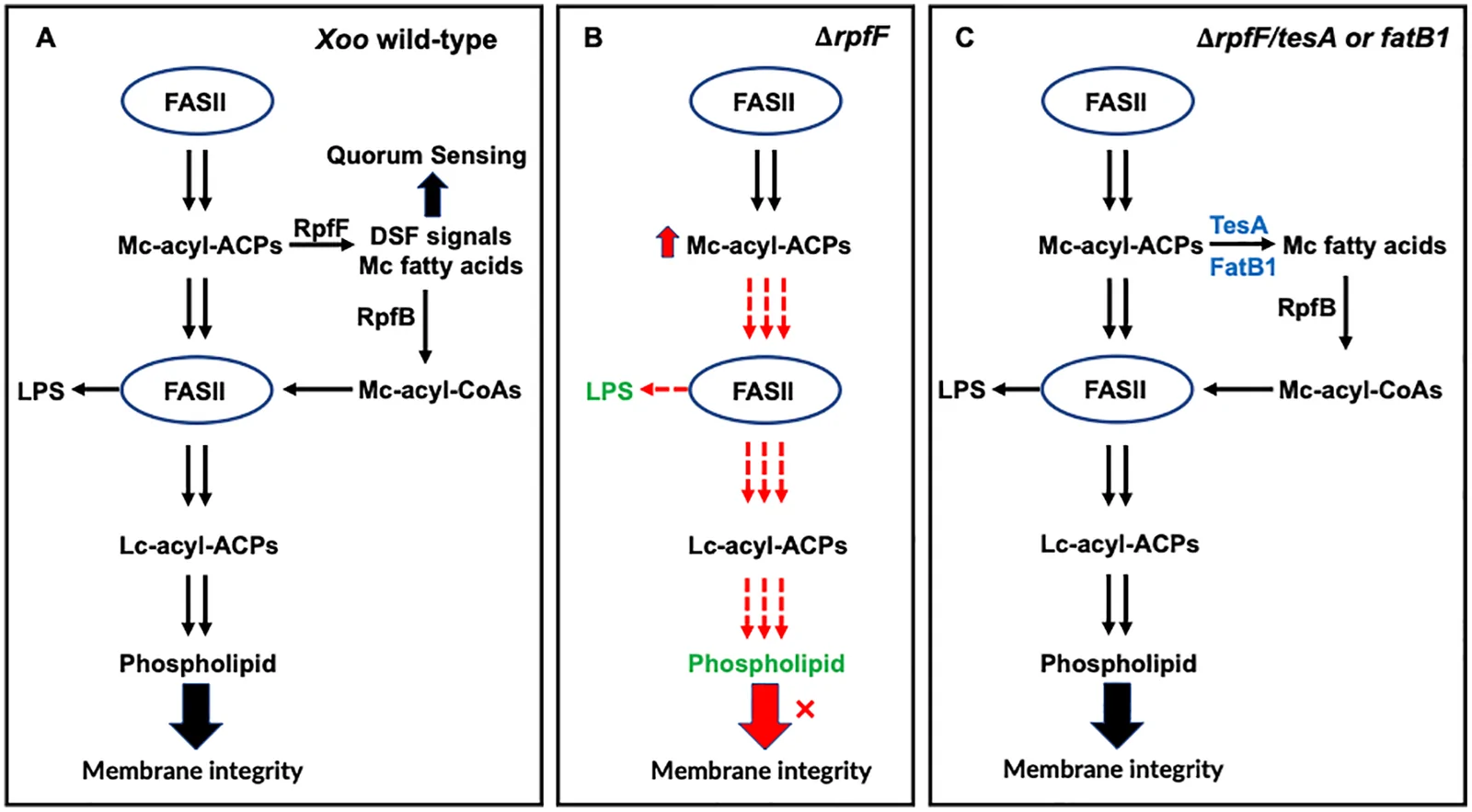
Working model for RpfF-mediated control of FASII flux and envelope homeostasis in *Xoo.* **(A) Wild type strain.** RpfF hydrolyses excess medium-chain (Mc) acyl-ACPs, keeping long-chain (Lc) acyl-ACP pools balanced for phospholipid and LPS synthesis; membrane integrity is preserved. **(B) ∆*rpfF* strain.** Mc-acyl-ACPs accumulate, FASII slows, Lc-acyl-ACP supply decreases, phospholipid/LPS biosynthesis is perturbed, and membrane integrity is compromised. **(C) ∆*rpfF* complemented with *EctesA* or *CcfatB1*.** Heterologous acyl-ACP thioesterases restore Mc-acyl-ACP consumption, rescue FASII flux, re-equilibrate Lc-acyl-ACP pools, and restore membrane integrity. FASII, fatty acid synthase type II system; Mc-acyl-ACPs or CoAs, medium-chain acyl-ACPs or CoAs; Lc-acyl-ACPs, long-chain acyl-ACPs; LPS, lipopolysaccharide.

The divergent phenotypic outcomes observed across *Xanthomonas* species underscore the known diversity of QS-regulated processes and highlight the importance of considering both the conserved thioesterase-dependent membrane maintenance function and the species-specific regulatory architecture of the DSF-dependent QS circuitry when interpreting *rpfF* mutant phenotypes.

## Materials and methods

### Materials

CoA, ATP, fatty acids, N-phenyl-1-naphthylamine (NPN), propidium iodide (PI), 3,3’-dipropylthiadicarbocyanine iodide (DiSC_3_(5)), and antibiotics were purchased from Sigma-Aldrich. Sodium [^14^C]acetate (57.0 mCi/mmol) was purchased from Amersham-Pharmacia Biotechnology. Molecular biology reagents were from Takara Bio, except for restriction endonucleases and T_4_ ligase, which were from New England Biolabs. Ni-agarose columns were obtained from Invitrogen. HC-C_18_ HPLC columns were provided by Agilent Technologies (Palo Alto, CA, United States). Oligonucleotides were synthesized by Takara Bio. Sangon Biotech (Shanghai) Co., Ltd provides the sequencing service. All other chemicals and reagents used were of the highest available quality.

### Bacterial strains, plasmids, and bacterial growth conditions

Bacterial strains and plasmids are listed in Supplementary Table A in S1 Appendix. *E. coli* were cultivated at 37 °C in LB medium (yeast extract, 5 g/L; peptone, 10 g/L; NaCl, 10 g/L; pH 7.0). *X*. *oryzae* pv. *oryzae* (*Xoo*) PXO99^A^ and its derived mutants were cultured at 30 °C in NA medium (beef extract, 3 g/L; yeast extract, 1 g/L; peptone, 5 g/L; sucrose,10 g/L; pH 7.0) [8]. When necessary, antibiotics and inducers were added to the cultures as follows: kanamycin sulfate, 30 µg/mL; gentamicin, 30 µg/mL; sodium ampicillin, 100 µg/mL. The final concentration of isopropyl-β-D-thiogalactoside (IPTG) was 1 mM.

### Recombinant DNA procedures and strain construction

All oligonucleotides are listed in Supplementary Table B in S1 Appendix. *Xoo rpfF* and *E. coli tesA* (lacking the 26-amino-acid signal peptide coding sequence) were amplified from genomic DNA of *Xoo* PXO99^A^ or *E. coli* MG1655 with Pfu DNA polymerase and ligated into pSRK-Gm [42] or pET-28b, respectively. *C*. *camphorum fatB1* was amplified from plasmid pXL49 [28] and inserted into the same vectors.

For p-mini-Tn7T-rpfF, two overlapping fragments were generated: the upstream segment contains PXO_0065, PXO_0066 and the 5′ portion of *rpfB*; the downstream segment contains the 3′ portion of *rpfB* plus the entire *rpfF* ORF (S1B Fig). After overlap-extension PCR, the fused product was cloned into pUC18T-mini-Tn7T-Gm [27]. p-mini-Tn7T-tesA and p-mini-Tn7T-fatB1 were assembled analogously, except that the P_*rpfF*_ promoter fragment was first amplified from p-mini-Tn7T-rpfF, fused to *tesA* or *fatB1* by overlap-extension PCR, and then inserted into pUC18T-mini-Tn7T-Gm. The above mini-Tn7 plasmids were electroporated into *Xoo* Δ*rpfF* or Δ*rpfB* Δ*rpfC* Δ*rpfF*, and integration at the *glmS* site was selected as described [26,27], yielding the strains: Δ*rpfF*::*rpfF*, Δ*rpfF*::*tesA*, Δ*rpfF*::*fatB1*, Δ*rpfB* Δ*rpfC* Δ*rpfF*::*rpfF*, Δ*rpfB* Δ*rpfC* Δ*rpfF*::*tesA*, Δ*rpfB* Δ*rpfC* Δ*rpfF*::*fatB1*.

### Growth and stress-tolerance assays

*Xoo* strains were cultivated in NA broth at 30 °C, and OD_600_ was monitored every 3 h for 6 days with a Bioscreen C automated analyzer (OY Growth Curves). For stress-tolerance assays, logarithmic-phase cultures were serially diluted, and 3 µL aliquots were spotted onto NA plates containing 0.0075% SDS, 0.1% Triton X-100, 1.5% NaCl, or 0.055 mM H_2_O_2_. After incubation at 30 °C for 3 days, colony growth was recorded.

### Electron microscopy

After 24 h of culture, the *rpfF* cells were harvested and washed twice with PBS. The pellet was resuspended in fresh PBS, and SDS was added to a final concentration of 0.01-0.02% (v/v). The mixture was gently mixed and incubated statically at 30 °C for 30 min. The cells were then collected and washed twice with PBS. For scanning electron microscopy, the pellet was resuspended in 2.5% glutaraldehyde fixative (40 × the pellet volume) and fixed at 4 °C for over 2 hours. The samples were rinsed 2–3 times with PBS buffer and dehydrated sequentially using 50%, 70%, and 100% ethanol (5–10 minutes each). The ethanol was replaced with an ethanol-tert-butanol mixture (1:1, v/v) for 20 minutes, followed by two changes of 100% tert-butanol (20 minutes each). After substitution, the samples were freeze-dried. Finally, the dried samples were coated using an ion sputter coater and observed/photographed under a scanning electron microscope. For transmission electron microscopy, the cells were negatively stained with 1% phosphotungstic acid (pH 7.0). A 3-µL droplet of the bacterial suspension was applied to a carbon-coated copper grid, excess liquid was blotted away after 3 min, and the grid was stained for 1 min. Excess stain was removed, the grid was air-dried, and examined with a Hitachi H-7650 TEM operated at 100 kV.

### Pathogenicity assays

Susceptible 40- to 45-day-old rice plants (*Oryza sativa* L. *japonica* cv. *Nipponbare*) were inoculated by the leaf-clipping method [7,22]. *Xoo* strains were grown to logarithmic phase in NA medium, harvested, washed, and resuspended in sterile PBS to 1 × 10^9^ CFU/ mL. Approximately 25 leaves per strain were clip-inoculated; lesion length was recorded at 14 dpi. For bacterial enumeration, 5–7 leaves per strain were collected at 1, 3, 5, 7, and 14 dpi, homogenized in PBS, serially diluted, and plated on NA. Data are means ± SEM of three independent experiments.

### EPS production, biofilm formation and motility

Qualitative EPS: 5 µL of logarithmic-phase cultures was spotted onto NA plates supplemented with 4% glucose and incubated 5 days at 30 °C; colony diameter was recorded. Quantitative EPS assay: log-phase cultures were shaken (200 rpm, 30 °C, 5 days) in NA medium supplemented with 4% glucose. EPS was precipitated with ice-cold absolute ethanol, dried, and weighed [7].

Biofilm: Glass tubes containing 3 mL NA were inoculated with logarithmic-phase cells and incubated statically 5 d at 30 °C. Planktonic cells were gently rinsed off; remaining biofilm was stained 10 min with 1% crystal violet, rinsed, and the dye solubilized in 1 mL 90% ethanol. Absorbance at 570 nm was measured to quantify biofilm biomass [7]. Cell aggregation: Logarithmic-phase cultures were left undisturbed 12 h at room temperature and scored visually [17].

Swimming motility: Cells grown in NA broth (30 °C, 2 d) were washed, resuspended in sterile PBS to OD_600_ of 2.0, and 2 µL aliquots were spotted onto the 0.3% agar NA plates. After 3 days at 30 °C the swim halo diameter was measured [17].

### Extracellular proteins analysis

*Xoo* strains were grown in 100 mL NA at 30 °C for 48 h. Cells were removed by centrifugation (4,000 rpm, 30 min, 4 °C), and the supernatant was brought to 50% (NH_4_)_2_SO_4_ saturation with slow stirring on ice. After overnight precipitation at 4 °C, proteins were pelleted (10,000 × g, 30 min, 4 °C), dissolved in 2 mL 50 mM ammonium acetate (pH 5.2), and dialyzed twice against 10 mM Tris-HCl (pH 8.0, 4 °C) in dialysis bag with a molecular weight cut-off of 10 kDa. Protein concentration was determined with the Bradford assay; aliquots were stored at -80 °C. Samples were separated on 12% SDS-PAGE and visualized by silver staining [22].

### Extracellular enzyme activity assays

Extracellular enzyme activities were assayed as described [17,22]. Cellulase and xylanase activities were determined by the 3,5-dinitrosalicylic acid (DNS) method. Reactions contained 2% (w/v) carboxymethyl cellulose (cellulase) or 1% (w/v) xylan (xylanase) in 50 mM sodium citrate buffer, pH 5.0. After incubation, the absorbance at 540 nm was measured. One unit of activity is defined as 1 µg of glucose (cellulase) or xylose (xylanase) equivalents released per minute per µg of total protein. Lipase activity was measured spectrophotometrically at 410 nm using p-nitrophenyl butyrate (pNPB) as substrate. One unit corresponds to 1 µg of p-nitrophenol liberated per minute per µg of protein.

### DSF family signals quantification

DSF family signals extracted and purified as described [43]. Extracts were analyzed on a Shimadzu LC-20AT HPLC fitted with a UV-220 detector and an Agilent C18 column (4.6 × 250 mm). An isocratic water/methanol mobile phase (23:77, v/v) containing 0.1% formic acid was run at 1 mL min^−1^; 20 µL of each sample was injected, and peaks were quantified by UV absorbance.

### Analysis of intracellular proteins leakage in *Xoo* strains

The pSRK-egfp plasmid was introduced into *Xoo* by electroporation. The transformants were selected and grown in liquid medium. Extracellular proteins were prepared as described previously. For intracellular proteins, pellets were lysed on ice for 2 h in 30 mM Tris-HCl (pH 8.0), 1 mM EDTA, 200 µg mL^−1^ lysozyme. Both fractions were analyzed by SDS-PAGE, and eGFP expression was detected by western blot with anti-FLAG antibody.

### Determination of membrane permeability in *Xoo* Strains

Outer-membrane permeability was assessed with the 1-N-phenylnaphthylamine (NPN) uptake assay [44]. *Xoo* cultures were grown in NA medium to OD_600_ of 1.0, harvested, and washed twice with 5 mM HEPES (pH 7.2) containing 20 mM glucose. Cells were resuspended to OD_600_ of 0.5, NPN was added to 10 µM, and fluorescence (with an excitation wavelength of 350 nm and an emission wavelength of 420 nm) was recorded every 5 min for 30 min in a microplate reader.

Inner-membrane permeability was evaluated with propidium iodide (PI) [45]. Cells were prepared as above, incubated with 10 nM PI for 30 min at 30 °C in the dark, and fluorescence was measured (excitation wavelength at 535 nm and emission wavelength at 615 nm).

Membrane depolarization was monitored with 3,3′-dipropylthiadicarbocyanine iodide (DiSC_3_(5)) [45]. Cultures (OD_600_ of 1.0) were washed twice with PBS, adjusted to OD_600_ of 0.5, treated with 10 nM DiSC_3_(5), and incubated for 30 min at 30 °C in the dark. Fluorescence was read at excitation wavelength 622 nm and emission wavelength 670 nm.

### Expression analysis by real-time RT-PCR

For RNA isolation, *Xoo* cells were grown in NA medium for 28 h at 28 °C at 200 rpm. RNA was isolated by Trizol (Invitrogen, CA, U.S.A.) method as the manufacturer’s instructions. Real-time RT-PCR was done as described previously [14].

### Analysis of phospholipid and fatty acid compositions

Phospholipid composition: *Xoo* strains were grown overnight in NA medium at 28 °C. [^14^C]acetate (5 µL, 2 µCi mL^−1^, 50–60 mCi mmol^−1^) was added and incubation continued for 12 h. Phospholipids were extracted, spotted on Silica gel G plates and developed in chloroform/methanol/acetic acid (65:25:8, v/v/v). Plates were air-dried and autoradiographed [46].

Fatty-acid analysis by GC-MS: Cells grown in NA medium (28 °C, 24 h) were subjected to lipid extraction [46]. The dried lipids were dissolved in 1.2 mL dry methanol and treated with 0.2 mL 25% (v/v) sodium methoxide for 15 min at room temperature. After addition of 1.2 mL 2 M HCl, fatty-acid methyl esters were extracted three times with 1.2 mL petroleum ether. The pooled extracts were dried under N_2_ and analyzed by GC-MS [47].

[^14^C]Acetate pulse-labelling of fatty-acid synthesis: *Xoo* was cultured in NA medium (28 °C, 16 h) and adjusted to identical OD_600_. Five millilitres of culture received 5 µL [^14^C]acetate (2 µCi mL^−1^, 50–60 mCi mmol^−1^) and was incubated for 1 h. Lipids were extracted, converted to methyl esters and separated by argentation-TLC prior to autoradiography [47].

Time-course measurement of fatty-acid synthesis: Cultures (10 mL) were grown to OD_600_ of 0.8, supplemented with 10 µL [^14^C]acetate (2 µCi mL^−1^, 50–60 mCi mmol^−1^) and incubated further. At fixed intervals, 2 mL aliquots were removed; lipids were extracted and redissolved in 50 µL hexane. Radioactivity was quantified by liquid-scintillation counting (Beckman Coulter). ^14^C incorporation into total lipids versus time served as the synthesis rate.

Extracellular free fatty acids: Strains were grown in 10 mL medium containing 10 µL [^14^C]acetate (2 µCi mL^−1^, 50–60 mCi mmol^−1^) for 24 h. Free fatty acids in the supernatant were extracted, derivatized to methyl esters, separated by argentation-TLC and visualized by autoradiography [25].

### Lipopolysaccharide (LPS) extraction of *Xoo* strains

*Xoo* strains were grown in NA medium to late log phase and adjusted to OD_600_ of 1.3. LPS was extracted from 1 mL of each cell pellet by the hot aqueous-phenol method [48]. Briefly, samples were treated with DNase I and RNase to remove nucleic acids, followed by proteinase K to eliminate proteins. After heating at 65 °C for 15 min in 90% phenol, the upper phase containing LPS was recovered by centrifugation. Twenty microlitres of each LPS preparation was resolved on a 12% SDS-PAGE and visualized by silver staining.

### *In vitro* assay of acyl-ACP thioesterase activity

Plasmids derived from pET-28b and encoding RpfF, TesA, or FatB1 were introduced into *E. coli* BL21(DE3). The corresponding His-tagged proteins were over-expressed and purified by Ni^2^^⁺^ -NTA chromatography as described [7]. *Vibrio harveyi* AasS (acyl-ACP synthetase), *Bacillus subtilis* Sfp (phosphopantetheinyl transferase), and *E. coli* apo-ACP were purified by the same protocol [7]. *holo*-ACP was prepared by incubating *apo*-ACP (100 µM) with CoA (200 µM) and Sfp (2 µM) in 50 mM Tris-HCl, pH 8.0, 10 mM MgCl_2_, 5 mM DTT at 37 °C for 3 h [7]. The product was purified and stored at -80 °C. Acyl-ACPs were generated in 50 mM Tris-HCl, pH 8.0, containing 100 µM *holo*-ACP, 2 mM ATP, and 1 mM fatty acid, with AasS (1 µM) at 30 °C for 1 h. Acyl-ACPs were purified and stored at -80 °C [49]. For qualitative acyl-ACP thioesterase assay, reactions (30 µL) contained 0.1 M Tris-HCl, pH 7.5, 2 mM β-mercaptoethanol, 20 µM acyl-ACP, and 0.2 µg purified enzyme (RpfF, TesA, or FatB1). After 30 min at 37 °C, products were resolved on 18% urea-PAGE gels and stained with Coomassie Brilliant Blue R-250. For quantitative acyl-ACP thioesterase assay, after 30 min at 37 °C, reaction mixture (480 µL) contained acyl-ACP thioesterase and acyl-ACP was quenched with 20 µL of 10 mM 5,5′-dithiobis (2-nitrobenzoic acid) in 0.1 M potassium phosphate, pH 8.0 [25,50]. Absorbance at 412 nm was recorded every 10 s. Activity is expressed as µmol free thiol released min^−1^ mg^−1^ protein, using ε_412_ = 14,150 M^−1^ cm^−1^.

## Supporting information

S1 Fig Genetic complementation of the *Xoo* Δ*rpfF* mutant. **(A) Strains construction**. The indicated acyl-ACP thioesterase gene was cloned into pUC18T-mini-Tn7T and delivered to the Δ*rpfF* mutant. Integration at the *glmS* locus was mediated by the Tn7 transposon system, yielding chromosomally complemented strains. **(B) Promoter architecture**. A 1.3-kb fragment (*P*_*rpfF*_) drives expression of the complementing gene. It comprises a 500-bp region upstream of *rpfB*, a truncated portion of *rpfB*, and the *rpfB-rpfF* intergenic sequence.
(ZIP)

S2 Fig Growth responses of *rpfF* strains to environmental stresses. **(A) Transmission electron microscopy of the *rpfF* strains after SDS treatment.** Collect the *rpfF* strains cultured for 24 h, wash it with PBS buffer, and resuspend it. Take an appropriate amount of the bacterial suspension, treat it with SDS for 30 min, and observe the sample by transmission electron microscopy. **(B) Growth responses of *rpfF* strains to H**_**2**_**O**_**2**_ **and NaCl.** *Xoo rpfF* strains cultures in the logarithmic phase were serially diluted and the dilution were inoculated onto NA plates containing H_2_O_2_ or NaCl followed by incubation at 30 °C for 3 days.
(ZIP)

S3 Fig Membrane permeability assessed by fluorescent dyes. **(A) Outer-membrane permeability was assessed by N-phenyl-1-naphthylamine (NPN) uptake.** Cells were resuspended in HEPES buffer, 10 µM NPN was added, and fluorescence was recorded every 5 min with a microplate reader. **(B) Inner-membrane permeability was determined with propidium iodide (PI).** After incubation with 10 µM PI for 30 min at 30 °C in the dark, fluorescence was measured. **(C) Inner-membrane depolarization was monitored with 3,3′-dipropylthiadicarbocyanine iodide (DiSC_3_(5)).** Cells in PBS were stained with 0.5 µM DiSC_3_(5) for 30 min at 30 °C in the dark before fluorescence measurement. The data represented mean values of three independent experiments.
(TIF)

S4 Fig qRT-PCR analysis of the genes involved in envelope stress response pathways. *htrA*, serine protease DegQ; *dsbC*, thiol:disulfide interchange protein (POX_02667); *hsp90*, molecular chaperone HtpG (PXO_01954); *rpoE*, RNA polymerase σ^70^ factor (PXO_00666). Data are presented as mean ± SD from n = 3 independent biological replicates. Statistical significance was determined using one-way ANOVA followed by Tukey’s multiple comparisons test. ****, *P* < 0.0001; ***, *P* < 0.001; **, *P* < 0.01; ns, not significant.
(TIF)

S5 Fig Virulence-associated traits of *rpfF* strains. **(A) Cell aggregation.** Logarithmic-phase cultures were left undisturbed 12 h at room temperature and photographed. **(B) Motility.** Bacterial halos formed on semisolid 0.3% agar plates after incubation at 30 °C for 2 days.
(TIF)

S6 Fig qRT-PCR analysis of phenotype-linked genes in *rpfF* strains. **(A) Genes involved in extracellular enzyme synthesis.** *engXCA*, endoglucanase (PXO_04151); *xynA*, endo-1,4-β-xylanase (PXO_03861). **(B) Genes involved in attachment.** *yapH*, methionine sulfoxide reductase catalytic subunit (PXO_00718); *xadA*, trimeric autotransporter adhesin (PXO_03061). **(C) Genes involved in chemotaxis.** *cheR*, chemotaxis protein methyltransferase (PXO_00055). **(D) Genes involved in exopolysaccharide synthesis.** *gumB*, polysaccharide biosynthesis/export protein (PXO_01391). Data are presented as mean ± SD from n = 3 independent biological replicates. Statistical significance was determined using one-way ANOVA followed by Tukey’s multiple comparisons test. ****, *P* < 0.0001; ***, *P* < 0.001; **, P < 0.01; ns, not significant.
(TIF)

S7 Fig (A) RpfF acyl-ACP thioesterase activity with acyl-ACPs of various chain lengths. C_4_-ACP, butyryl-ACP; C_6_-ACP, hexanoyl-ACP; C_8_-ACP, octanoyl-ACP; C_10_-ACP, decanoyl-ACP; C_12_-ACP, dodecanoyl-ACP; C_14_-ACP, tetradecanoyl-ACP. Data in A is presented as mean ± SD from n = 3 independent biological replicates. Statistical significance was determined using one-way ANOVA followed by Tukey’s multiple comparisons test. **, *P* < 0.01; *, *P* < 0.05; ns, not significant. **(B) Production of DSF family signals in *rpfF* strains.**
(ZIP)

S8 Fig Deletion of *rpfF* in *Xcc* disrupt membrane integrity. **(A)** Growth responses of *Xcc rpfF* strains to detergents. **(B)** Thin-layer chromatography (TCL) analysis of the phospholipids extracted from *Xcc rpfF* strains. **(C)** Lipopolysaccharide from *Xcc rpfF* strains separated by SDS-PAGE.
(ZIP)

S9 Fig RpfC inhibits the acyl-ACP thioesterase activity of RpfF in *Xoo.* **(A)** Overexpression of *rpfC* renders *Xoo* sensitive to Triton X-100. **(B)** RpfC REC domain inhibits the acyl-ACP thioesterase activity of RpfF *in vitro*. REC, RpfC receiver domain.
(ZIP)

S1 Appendix Table A. Bacterial strains and plasmids. **Table B.** Sequences of PCR primers. **Table C.** Scintillation counting determination of *rpfF* strains phospholipids and lipid A contents.
(DOCX)

S1 File Raw data. (XLSX)

S2 File Rawgel. (PDF)
