# Supplementary material for: The RpfF Acyl-ACP thioesterase of major rice pathogen Xanthomonas oryzae pv. oryzae is essential for maintenance of membrane function and virulence
Source: PLoS Pathog. 2026 Oct 6;22(10):e1014669. doi: 10.1371/journal.ppat.1014669 (PMC13660987; doi:10.1371/journal.ppat.1014669)
Supplement: Supplementary file 1 [file ppat.1014669.s012.pdf]

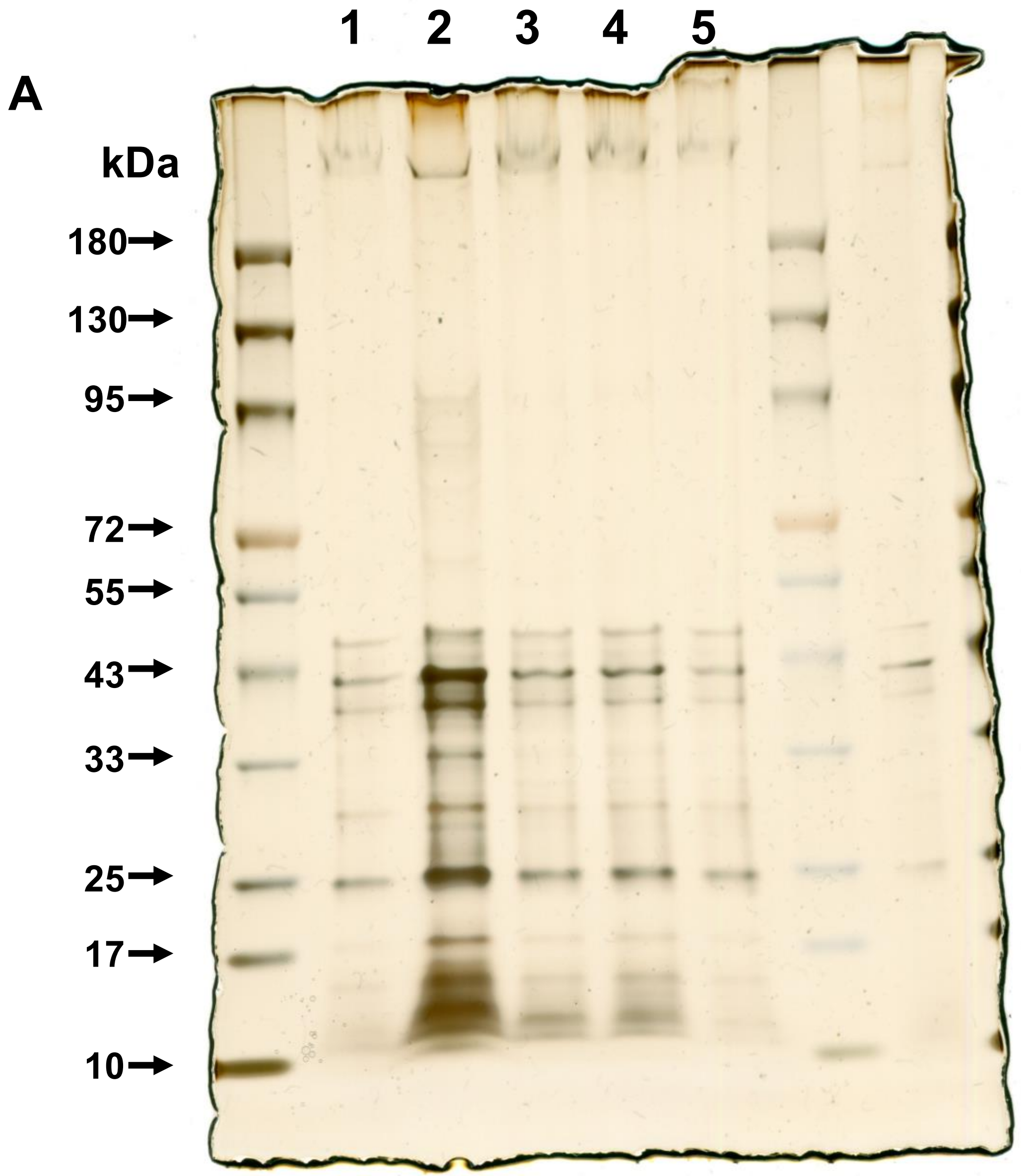
A
1
2
3
4
5
kDa
180
130
95
72
55
43
33
25
17
10

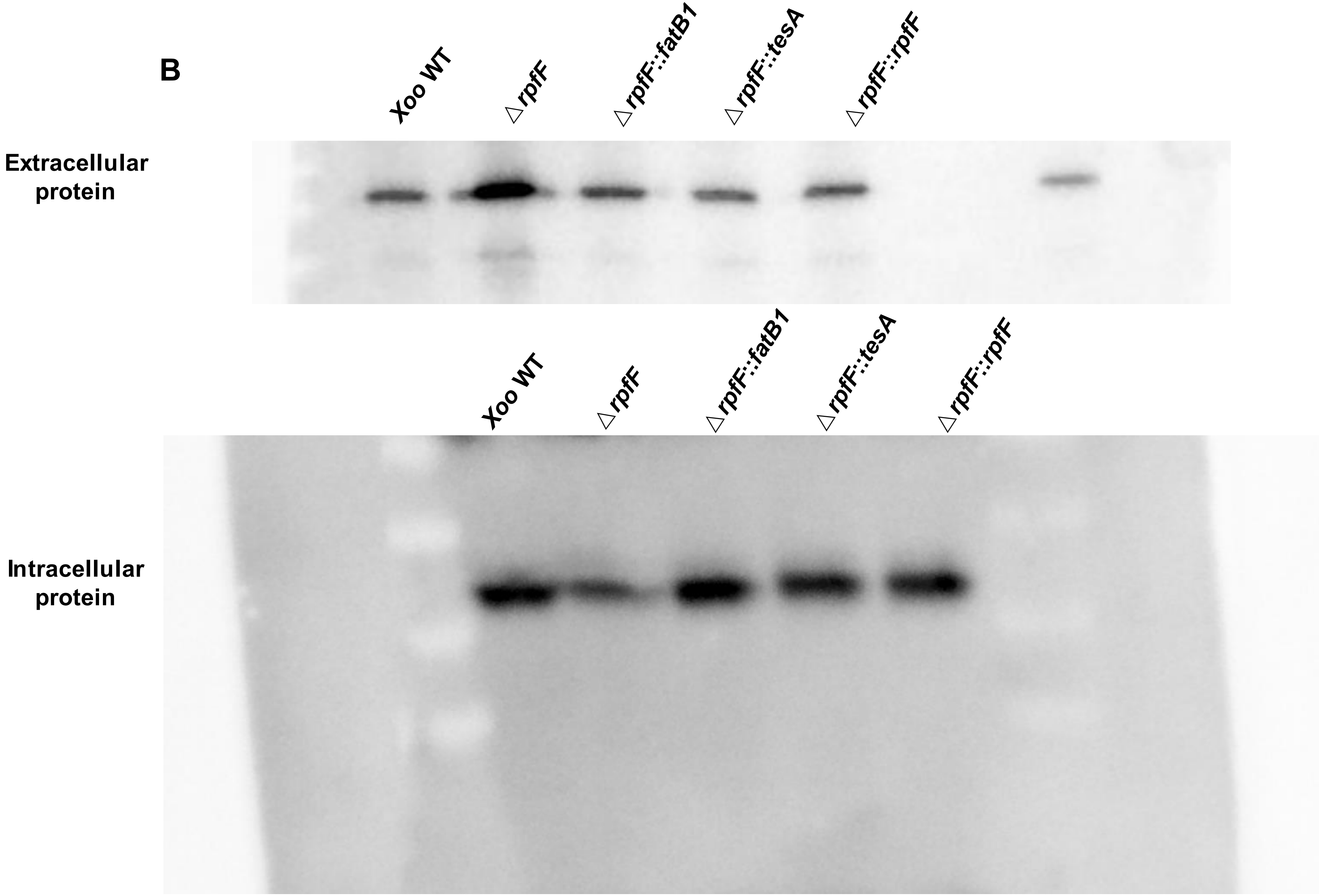

B
Xoo WT
△rpfF
△rpfF::fatB1
△rpfF::tesA
△rpfF::rpfF
Extracellular protein
Xoo WT
△rpfF
△rpfF::fatB1
△rpfF::tesA
△rpfF::rpfF
Intracellular protein

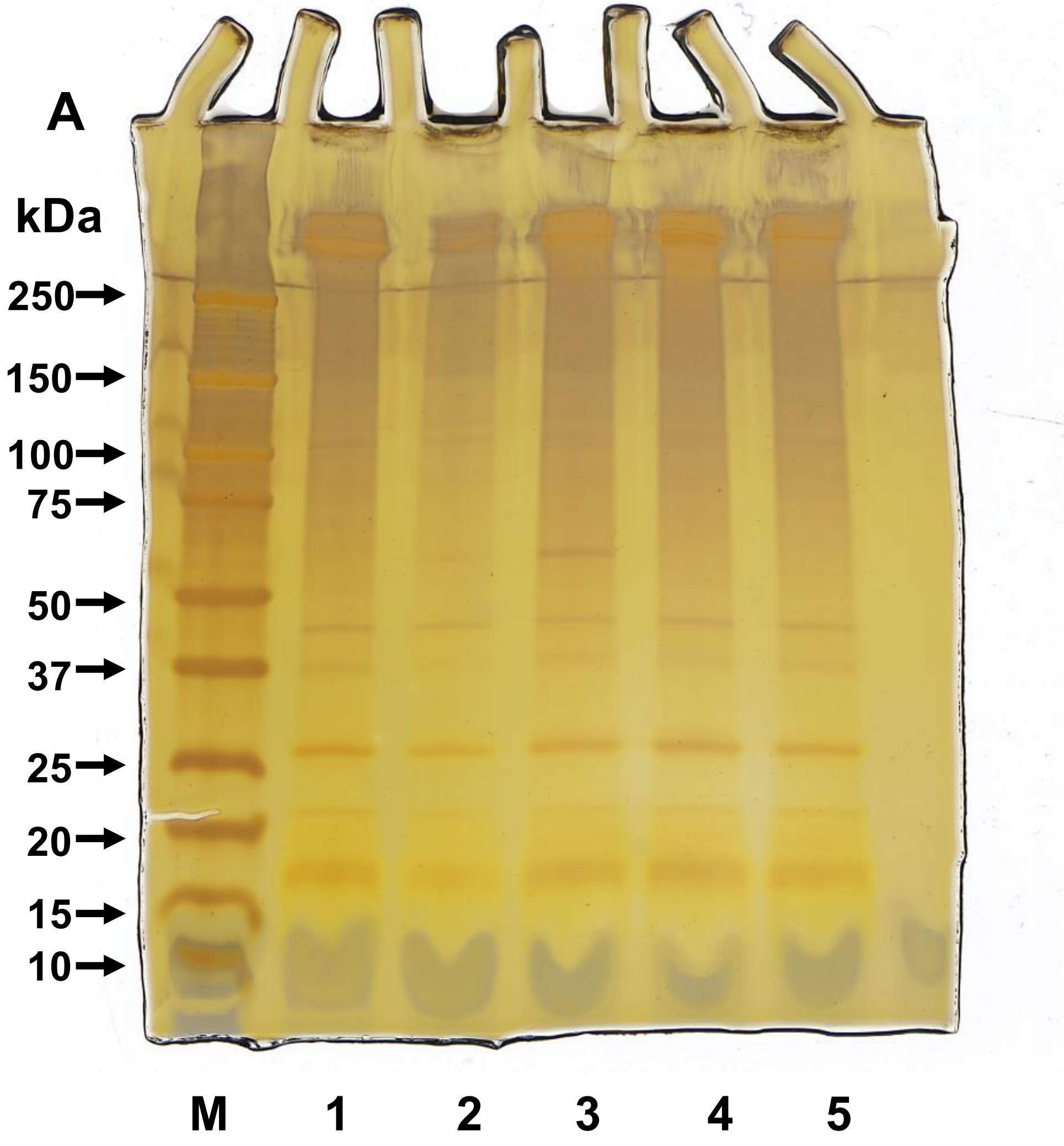
A
kDa
250
150
100
75
50
37
25
20
15
10
M
1
2
3
4
5

**A**

| | | | | | | | | | | |
|---|---|---|---|---|---|---|---|---|---|---|
| $C_4$-ACP | + | + | + | + | - | - | - | - | - | - |
| $C_6$-ACP | - | - | - | - | - | + | + | + | + | - |
| RpfF | - | + | - | - | - | - | + | - | - | - |
| TesA | - | - | + | - | - | - | - | + | - | - |
| FatB1 | - | - | - | + | - | - | - | - | + | - |

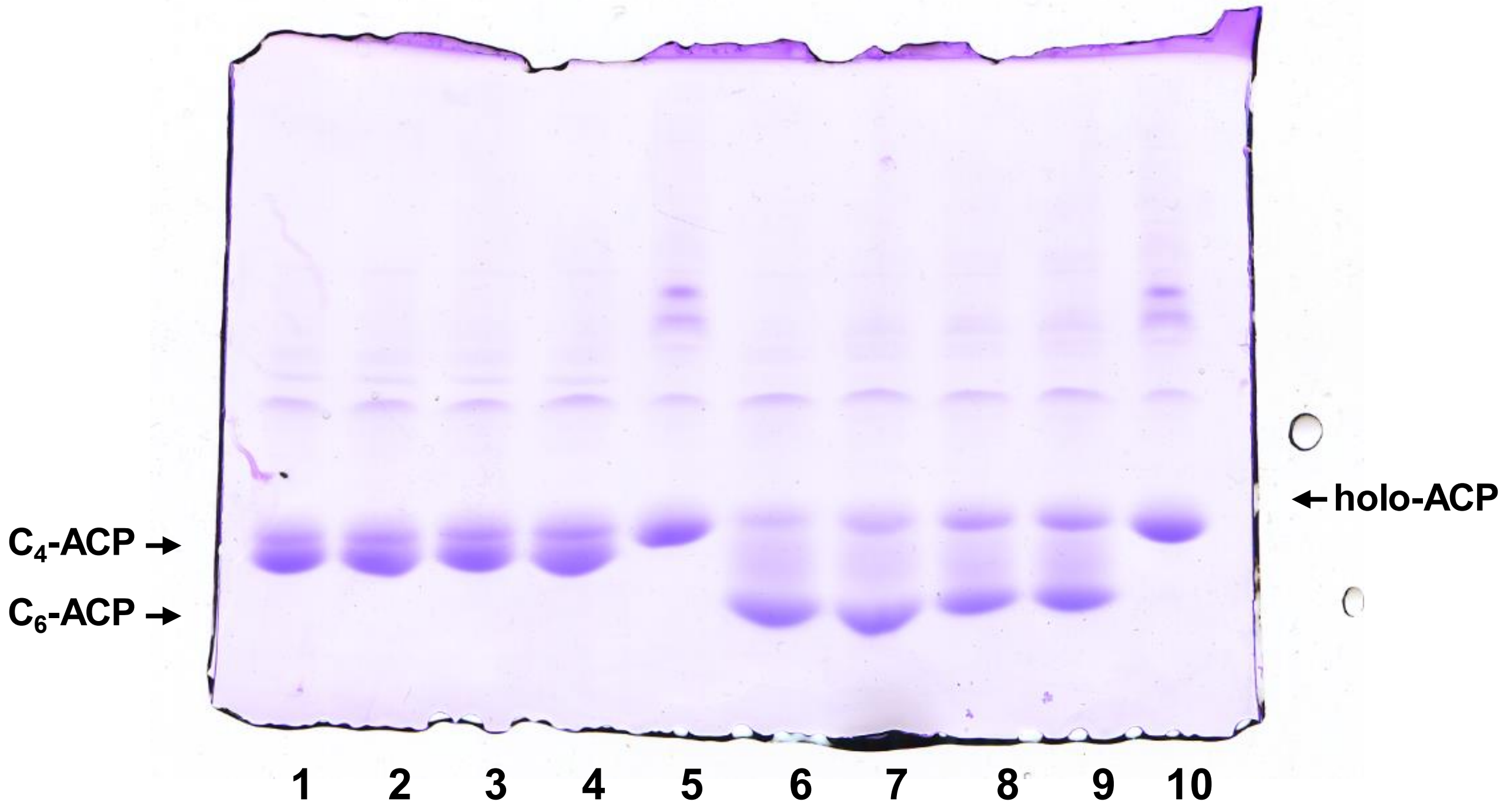

**B**

| | | | | | | | | | | |
|---|---|---|---|---|---|---|---|---|---|---|
| $C_8$-ACP | + | + | + | + | - | - | - | - | - | - |
| $C_{10}$-ACP | - | - | - | - | - | + | + | + | + | - |
| RpfF | - | + | - | - | - | - | + | - | - | - |
| TesA | - | - | + | - | - | - | - | + | - | - |
| FatB1 | - | - | - | + | - | - | - | - | + | - |

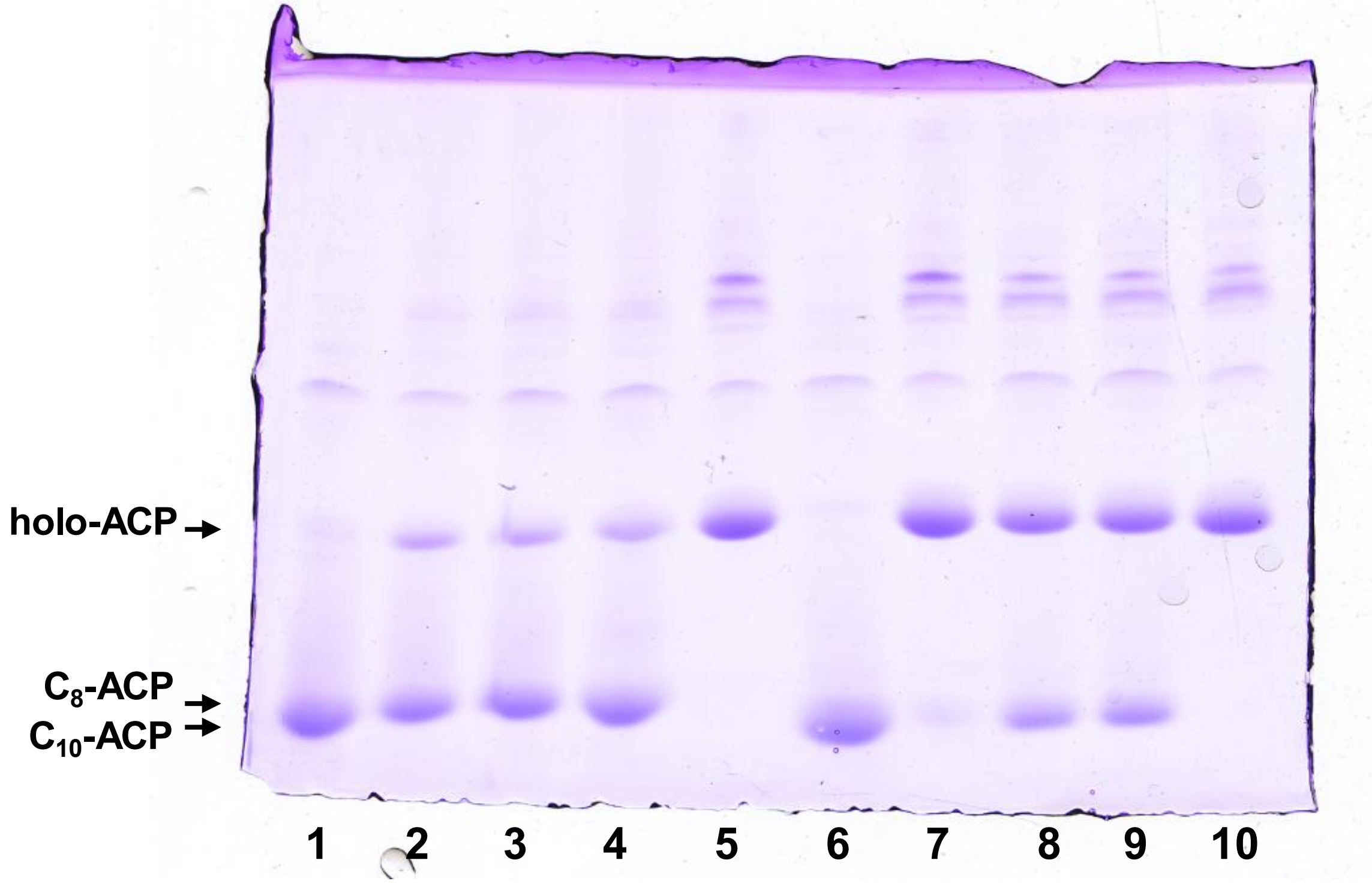

**C**

| | | | | | | | | | | |
|---|---|---|---|---|---|---|---|---|---|---|
| $C_{12}$-ACP | - | + | + | + | + | - | - | - | - | - |
| $C_{14}$-ACP | - | - | - | - | - | - | + | + | + | + |
| RpfF | - | + | - | - | - | - | + | - | - | - |
| TesA | - | - | + | - | - | - | - | + | - | - |
| FatB1 | - | - | - | + | - | - | - | - | + | - |

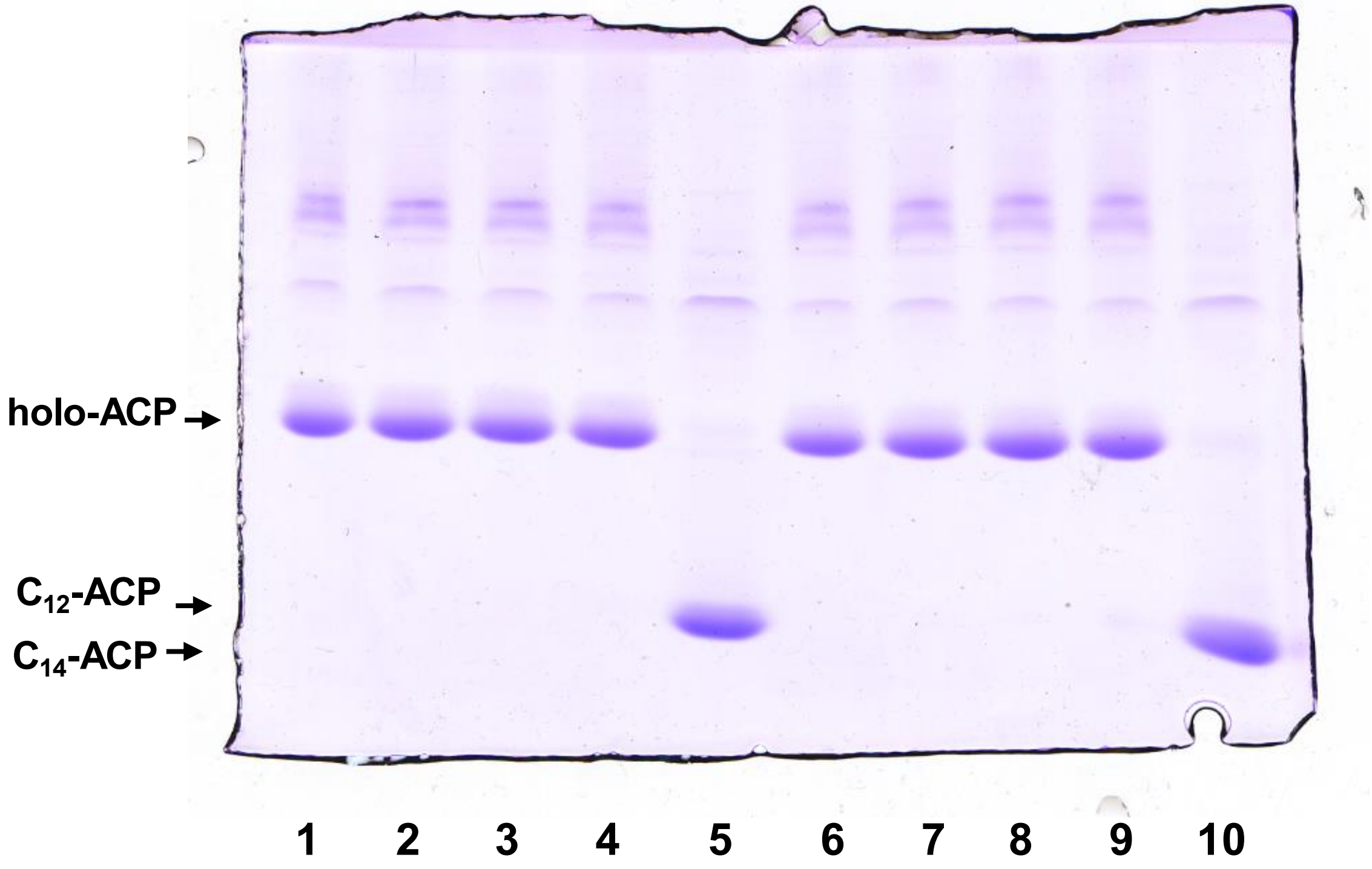

C

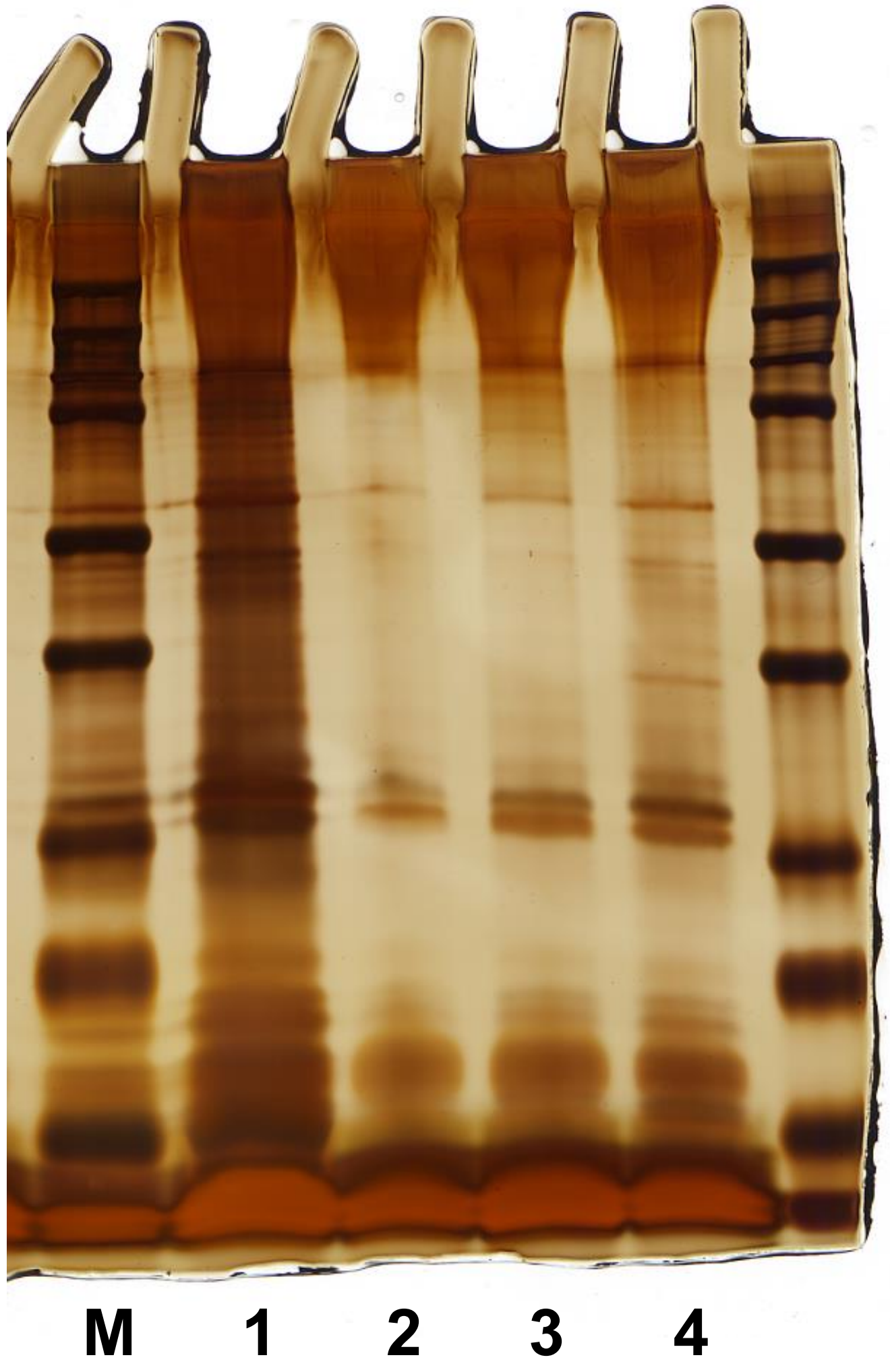

**B**

| | | | | | |
|---|---|---|---|---|---|
| $C_{12}$-ACP | - | + | + | + | + |
| RpfF | - | - | + | + | - |
| RpfC-REC | - | - | + | - | - |

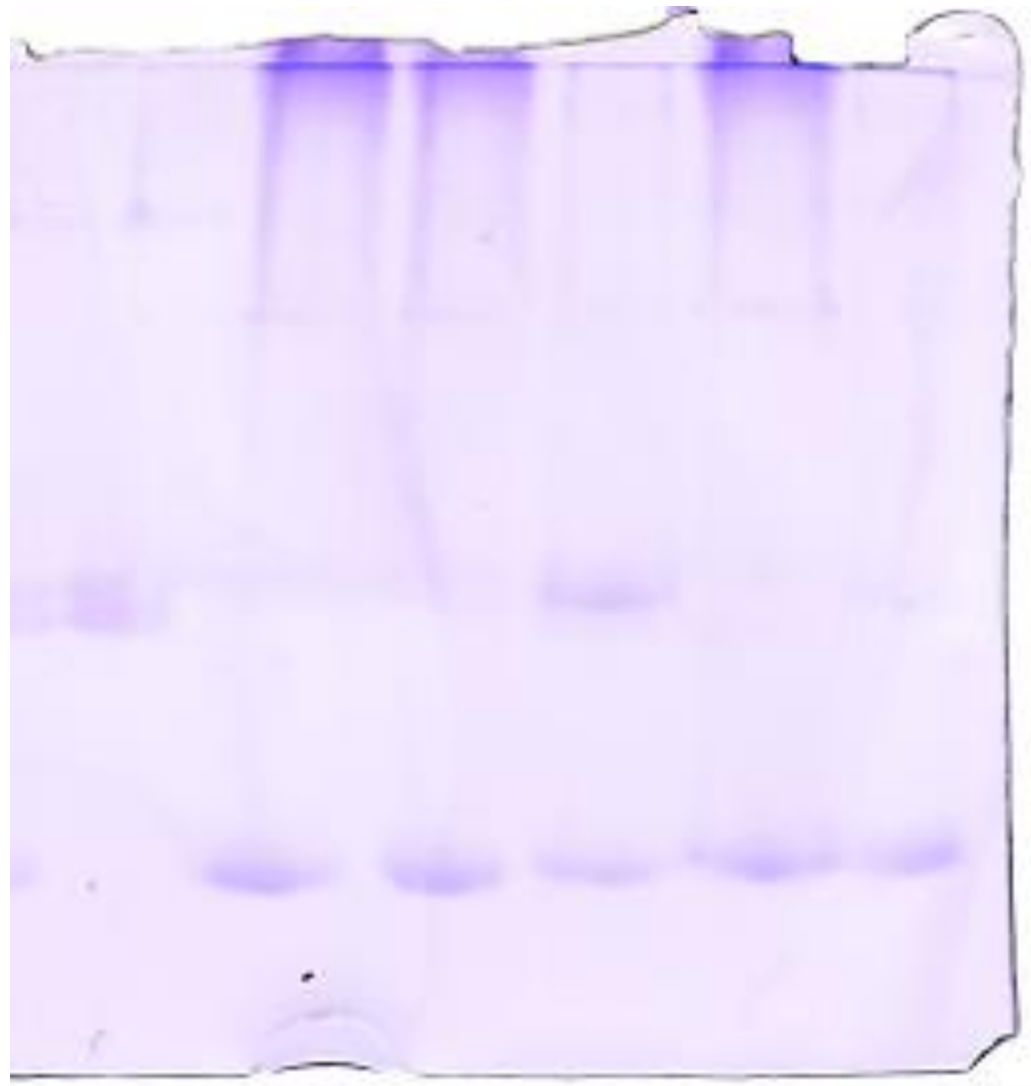

holo-ACP →

1 2 3 4 5
