# Supplementary material for: The RpfF Acyl-ACP thioesterase of major rice pathogen Xanthomonas oryzae pv. oryzae is essential for maintenance of membrane function and virulence
Source: PLoS Pathog. 2026 Oct 6;22(10):e1014669. doi: 10.1371/journal.ppat.1014669 (PMC13660987; doi:10.1371/journal.ppat.1014669)
Supplement: Supplementary file 2 [file ppat.1014669.s010.docx]

Supplementary Materials for

**The RpfF Acyl-ACP Thioesterase of Major Rice Pathogen *Xanthomonas* *oryzae* is Essential for Maintenance of Membrane Function and Virulence**

Lizhen Luo *et al.*

*Corresponding author. Email: wanghh36@scau.edu.cn or jecronan@illinois.edu

**This file includes:**

Table A to C

Table A. Bacterial strains and plasmids

| Strains or Plasmids | Relevant characteristic(s) | Source or reference |
| --- | --- | --- |
| **Strains** |  |  |
| ***E. coli*** |  |  |
| DH5α | F’ ϕ 80∆*lacZ*∆M15/∆(lac*ZYA*-*argF*)*U169* *recA1* *endA1* *hsdR17* | Lab collection |
| BL21(DE3) | *E. coli* B F^-^ *ompT* r_B_^-^ m_B_^-^ (λDE3) | Lab collection |
| JW1794 | *E. coli* BW25113 Δ*fadD* Δ*araBAD* strain |  |
| ***Xoo*** |  |  |
| PXO99^A^ | Wild type | Lab collection |
| ∆*rpfF* | Deletion of *rpfF* in strain PXO99^A^ | Lab collection |
| ∆*rpfC* | Deletion of *rpfC* in strain PXO99^A^ | Lab collection |
| ∆*rpfBrpfC* | Deletion of *rpfB* and *rpfC* in strain PXO99^A^ | Lab collection |
| ∆*rpfBrpfCrpfF* | Deletion of *rpfB*, *rpfC* and *rpfF* in strain PXO99^A^ | Lab collection |
| ∆*rpfF*::*rpfF* | P*_rpfF_-rpfF* was inserted into *glmS* locus of ∆*rpfF* mutant by Tn7 transposon system | This study |
| ∆*rpfF*::*tesA* | P*_rpfF_-tesA* was inserted into *glmS* locus of ∆*rpfF* mutant by Tn7 transposon system | This study |
| ∆*rpfF*::*fatB1* | P*_rpfF_-fatB1* was inserted into *glmS* locus of ∆*rpfF* mutant by Tn7 transposon system | This study |
| ∆*rpfBrpfCrpfF*::*rpfF* | P*_rpfF_-rpfF* was inserted into *glmS* locus of ∆*rpfBrpfCrpfF* mutant by Tn7 transposon system | This study |
| ∆*rpfBrpfCrpfF*::*tesA* | P*_rpfF_-tesA* was inserted into *glmS* locus of ∆*rpfBrpfCrpfF* mutant by Tn7 transposon system | This study |
| ∆*rpfBrpfCrpfF*::*fatB1* | P*_rpfF_-fatB1* was inserted into *glmS* locus of ∆*rpfBrpfCrpfF* mutant by Tn7 transposon system | This study |
| **Plasmids** |  |  |
| pSRK-Gm | broad-host-range expression vector containing *lac* promoter and *lacI*^q^, *lacZα*^+^, and Gm^r^ |  |
| pET28(b**)** | Km^r^, expression vector in *E. coli* BL21(DE3) | Lab collection |
| pUC18T-mini-Tn7T-Gm | Amp^r^, Gm^r^, a versatile Mini-Tn7 delivery vector |  |
| pXL49 | Km^r^, *C. camphorum fatB1* cloned into pET28(b) |  |
| pET28b-Sfp | Km^r^, *B. subtilis* sfp cloned into pET28(b) |  |
| pYFJ84 | Amp^r^, *V. harveyi* aasS cloned in pET16(b) |  |
| pET28b-ACP | Km^r^, *E. coli acpP* cloned in pET28(b) with NdeI and HindIII |  |
| pET28-RpfF | *Xoo rpfF* cloned into pET28(b) with NdeI and HindIII | This study |
| pET28-TesA | *E. coli tesA* cloned into pET28(b) with NdeI and HindIII | This study |
| pET28-FatB1 | *C. camphorum fatB1* cloned into pET28(b) with NdeI and HindIII | This study |
| pSRK-egfp | *egfp-flag* fusion gene cloned into vector pSRK-Gm with NdeI and HindIII, Gm^r^ | Lab collection |
| p-mini-Tn7T-rpfF | *Xoo rpfF* cloned in vector pUC18T-mini-Tn7T-Gm with SpeI and HindIII, under the control of P*_rpfF_*, Gm^r^ | This study |
| p-mini-Tn7T-tesA | *E. coli tesA* cloned in vector pUC18T-mini-Tn7T-Gm with SpeI and HindIII, under the control of P*_rpfF_*, Gm^r^ | This study |
| p-mini-Tn7T-fatB1 | *C. camphorum fatB1* cloned in vector pUC18T-mini-Tn7T-Gm with SpeI and HindIII, under the control of P*_rpfF_*, Gm^r^ | This study |
| pSRK-rpfF | *Xoo rpfF* cloned into vector pSRK-Gm with SacI and HindIII, Gm^r^ | This study |
| pSRK-tesA | *E. coli tesA* cloned in vector pSRK-Gm with NdeI and HindIII, Gm^r^ | This study |
| pSRK-fatB1 | *C. camphorum fatB1* cloned in vector pSRK-Gm with NdeI and HindIII, Gm^r^ | This study |
| pSRK-rpfC | Gm^r^, *rpfC* was inserted into pSRK-Gm | This study |
| pSRK-REC | Gm^r^, the REC domain DNA fragment of RpfC was inserted into pSRK-Gm | This study |

Table B. Sequences of PCR primers

| Primer name | Primer sequence (5′ to 3′) | Digestion sites^a^ |
| --- | --- | --- |
| P*xoorpfF Spe*I P1 | CGGactagttcaaaccgaatgacttggc | *Spe*I |
| P*xoorpfF* P2 | gaagccttgatactgcaggcaattgggc |  |
| P*xoorpfF* P3 | gcccaattgcctgcagtatcaaggcttcgtctacatcgtc |  |
| P*xoorpfF* P4 | catgttggttctccgtgatg |  |
| Tn7-*xoorpfF Hin*dIII | CCCAAGCTTtcagccggcgtcaagcc | *Hin*dIII |
| Tn7*-EctesA* F | catcacggagaaccaacATGGCGGACACGTTA |  |
| Tn7*-EctesA* R | cagcccgggggatccTTATGAGTCATGATTTAC | BamHI |
| Tn7*-*pXL49 F | catcacggagaaccaacATGGCGACTACTTCTCTG |  |
| Tn7*-*pXL49 *Hin*dIII R | CCCaagcttTTAAACGCTGGACTCAGC | *Hin*dIII |
| *XoorpfC REC Nde*I F | CACAGGAAACAGCATATGAACCCGTTTCTGCGTCATC | *Nde*I |
| *XoorpfC REC Hin*dIII R | CGACGGTATCGATAAGCTTctaACTGGTCGGTGCCTGC | *Hin*dIII |
| *XoorpfC Nde*I F | CACACAGGAAACAGCATATGAAGTCTCCACTGACATGG | *Nde*I |
| *XoorpfC Nde*I F | CACACAGGAAACAGCATATGAAGTCTCCACTGACATGG | *Nde*I |
| *pETrpfF Nde*I F | GGTGCCGCGCGGCAGCCATATGatgtctgcagttcaacc | *Nde*I |
| *pETrpfF Hin*dIII R | CGAGTGCGGCCGCAAGCTTgccggcgtcaagcccggag | *Hin*dIII |
| pXL49 *Nde*I F | GGTGCCGCGCGGCAGCCATATGGCGACTACTTCTCTG | *Nde* I |
| pXL49 *Hin*dIII R | CGAGTGCGGCCGCaagcttTTAAACGCTGGACTCAGC | *Hin*d III |
| *EctesA Nde*I F | CCGCGCGGCAGCCATATGATGGCGGACACGTTATTG | *Nde*I |
| *EctesA Hin*dIII R | CGAGTGCGGCCGCAAGCTTTGAGTCATGATTTACTAAAG | *Hin*d III |
| *xoorpfF Sac*I | CCGAGCTCtatgtctgcagttcaaccc | *Sac*I |
| *xoorpfF Hin*dIII | CCCAAGCTTtcagccggcgtcaagcc | *Hin*d III |
| *fliC* F | ATGCAGGAAATCGGCAACAA |  |
| *fliC* R | TTGAAGCTGGTCTGATTGGC |  |
| *flgA* F | CACCAATACGGTGGAAGTGG |  |
| *flgA* R | GCATCACGCTTCTCGATACT |  |
| *fliQ* F | CTGAAATCGCCCTGACTGAA |  |
| *fliQ* R | CAATGGTCGGCTCGTTCAA |  |
| *cheR* F | ACGTCGATACCCAAGTGTTG |  |
| *cheR* R | TGGCGGTATTCGATGAGTTG |  |
| *engXCA* F | ATGATCAACCAGATGCAGGG |  |
| *engXCA* R | TCGTTGATCACCTTGTCGAG |  |
| *XynA* F | TGCAGTTCCAGTTCCATGTC |  |
| *XynA* R | ATTGGCCACCTGCATCAAAT |  |
| *gumB* F | GATCTGCTCGATGACACTGG |  |
| *gumB* R | TCTGGAACACCTTGAACAGC |  |
| *yapH* F | GATTCTGAGTTCGGACACCC |  |
| *yapH* R | ATCGAAACACTGCCGTTGAT |  |
| *XadA* F | TTCTGCCTGACCATGTAACG |  |
| *XadA* R | TGGTGTGTGCAGTTGCCAGA |  |
| *rpoE* F | CGCAGAAGGAGAACAGGTC |  |
| *rpoE* R | TCCCGATACGACAACCCTTC |  |
| *htrA* F | GACAACCTCACCGACATCAA |  |
| *htrA* R | GAAGTTCTGATAGCCCAGCC |  |
| *dsbC* F | CCGTTCGACATCCAGAACAA |  |
| *dsbC* R | CCACATTCCACGTCGGTAAA |  |
| *Hsp90* F | ATCCAGAATCACATGCACCG |  |
| *Hsp90* R | GCATCCTGCTGCGTCATAA |  |
|  |  |  |

*^a^* Underlined nucleotide sequences are digestion sites of restriction endonucleases.

**Table C. Scintillation counting determination of *rpfF* strains phospholipids and lipid A contents**

|  | Phospholipids  (cpm) | Lipid A  (cpm) | Lipid A/phospholipids |
| --- | --- | --- | --- |
| *Xoo* WT | 4525.67 ± 90.51 | 531.61 ± 23.36 | 0.117 |
| *rpfF* | 9197.11 ± 392.56 | 337.50 ± 13.03 | 0.037 |
| *rpfF*::*fatB1* | 8082.54 ± 214.17 | 510.54 ± 5.84 | 0.06 |
| *rpfF*::*tesA* | 3288.61 ± 21.44 | 576.38 ± 15.36 | 0.175 |
| *rpfF*::*rpfF* | 4657 ± 168.76 | 619.22 ± 5.54 | 0.133 |
